# DNA Damage Response Factors from Diverse Pathways, Including DNA Crosslink Repair, Mediate Alternative End Joining

**DOI:** 10.1371/journal.pgen.1004943

**Published:** 2015-01-28

**Authors:** Sean M. Howard, Diana A. Yanez, Jeremy M. Stark

**Affiliations:** 1 Department of Radiation Biology, Beckman Research Institute of the City of Hope, Duarte, California, United States of America; 2 Irell and Manella Graduate School of Biological Sciences, Beckman Research Institute of the City of Hope, Duarte, California, United States of America; University of Washington School of Medicine, UNITED STATES

## Abstract

Alternative end joining (Alt-EJ) chromosomal break repair involves bypassing classical non-homologous end joining (c-NHEJ), and such repair causes mutations often with microhomology at the repair junction. Since the mediators of Alt-EJ are not well understood, we have sought to identify DNA damage response (DDR) factors important for this repair event. Using chromosomal break reporter assays, we surveyed an RNAi library targeting known DDR factors for siRNAs that cause a specific decrease in Alt-EJ, relative to an EJ event that is a composite of Alt-EJ and c-NHEJ (Distal-EJ between two tandem breaks). From this analysis, we identified several DDR factors that are specifically important for Alt-EJ relative to Distal-EJ. While these factors are from diverse pathways, we also found that most of them also promote homologous recombination (HR), including factors important for DNA crosslink repair, such as the Fanconi Anemia factor, FANCA. Since bypass of c-NHEJ is likely important for both Alt-EJ and HR, we disrupted the c-NHEJ factor Ku70 in Fanca-deficient mouse cells and found that Ku70 loss significantly diminishes the influence of Fanca on Alt-EJ. In contrast, an inhibitor of poly ADP-ribose polymerase (PARP) causes a decrease in Alt-EJ that is enhanced by Ku70 loss. Additionally, the helicase/nuclease DNA2 appears to have distinct effects from FANCA and PARP on both Alt-EJ, as well as end resection. Finally, we found that the proteasome inhibitor Bortezomib, a cancer therapeutic that has been shown to disrupt FANC signaling, causes a significant reduction in both Alt-EJ and HR, relative to Distal-EJ, as well as a substantial loss of end resection. We suggest that several distinct DDR functions are important for Alt-EJ, which include promoting bypass of c-NHEJ and end resection.

## Author Summary

Alternative EJ (Alt-EJ) is a chromosomal double strand break (DSB) repair pathway that often uses short stretches of homology (microhomology) to bridge the break during repair. Alt-EJ involves bypass of the classical non-homologous end joining (c-NHEJ) pathway, and hence may be important for DSBs that are not readily repaired by c-NHEJ, such as DSBs requiring extensive end processing prior to ligation. Since the factors that mediate Alt-EJ are unclear, we identified DNA damage response factors that differentially promote Alt-EJ relative to an EJ event that is a composite of c-NHEJ and Alt-EJ. Several of these factors promote other repair events that are enhanced by loss of c-NHEJ, namely homologous recombination (HR), including DNA crosslink repair factors, such as FANCA. We then investigated distinctions among individual factors. For instance, we found that loss of c-NHEJ appears to diminish the influence of FANCA on Alt-EJ, but enhances the effect of PARP inhibition. Furthermore, we find that FANCA and DNA2 differentially affect another aspect of the DNA damage response, namely end resection. Based on these findings, we suggest that several aspects of the DNA damage response are important for Alt-EJ.

## Introduction

End joining (EJ) repair of chromosomal breaks is important for cellular resistance to clastogens, and for antibody maturation that is induced by programmed double-strand breaks (DSBs) [1]. However, EJ can be prone to cause loss of genetic information, as it does not require the use of extensive homology or a template for repair. Loss of genetic information can include insertions or deletions, point mutations, and/or formation of gross chromosomal rearrangements. Such gross chromosomal rearrangements are associated with cancer and inherited diseases, and can often show evidence of short stretches of homology (microhomology) at the rearrangement junctions [2–4]. Defining the factors that influence the frequency of these different EJ outcomes provides insight into the processes that ensure genome maintenance.

Repair via EJ can be classified into two major types: classical non-homologous EJ (c-NHEJ) and alternative-EJ (Alt-EJ) [5–8]. C-NHEJ events are mediated by a set of factors important for antibody maturation, including the DSB end binding factor Ku (Ku70/80 heterodimer), the kinase DNA-PKcs, and the XRCC4/Ligase 4 complex [1]. These c-NHEJ factors are also important for radioresistance, yet in their absence, chromosomal EJ remains relatively proficient, but repair junctions show increased frequencies of insertions and deletions, as well as greater evidence of microhomology usage [9–11]. The term Alt-EJ refers to such repair events that are independent of c-NHEJ factors [9–13]. While Alt-EJ events often show microhomology at the repair junction, microhomolgy is not absolutely essential for Alt-EJ [9–13]. Furthermore, c-NHEJ can also use microhomolgy during repair [14].

The increase in Alt-EJ caused by loss of c-NHEJ is a feature shared with homologous recombination (HR). Namely, at least two types of HR are more frequent in the absence of c-NHEJ factors: the conservative homology-directed repair (HDR) pathway that is mediated by the strand invasion factor RAD51, as well as the non-conservative single-stranded annealing (SSA) pathway [15, 16]. Since loss of c-NHEJ causes a substantial increase in the frequency of HDR, SSA, and Alt-EJ, bypass of c-NHEJ is likely an important step of these repair events. Although, such c-NHEJ bypass may not be an absolute requirement, since c-NHEJ is not necessarily the default pathway of DSB repair in all circumstances, such as distinct chromatin and cell cycle phase contexts. Nevertheless, c-NHEJ bypass likely enables DSB end resection that generates 3’ ssDNA. Accordingly, factors important for end resection, including CtIP and the Mre11-complex, mediate HDR, SSA, and Alt-EJ [12, 17–23]. The role of these factors is conserved in *S. cerevisiae*, in that the CtIP ortholog (*SAE2*) and *MRE11* are important for Alt-EJ/MMEJ in this organism [24]. In contrast, factors that are implicated in promoting extensive end resection (e.g. BLM and EXO1) appear to favor HR over Alt-EJ [18, 25]. Conversely, factors that inhibit end resection, such as 53BP1 and RIF1, suppress HDR, SSA, and Alt-EJ [25–28].

Such inhibition of c-NHEJ to enable chromosomal break end resection for HR and/or Alt-EJ has implications for genome maintenance beyond DSB repair outcome. Namely, chromosomal breaks that occur at DNA replication forks can be one-ended DSBs, which if used during c-NHEJ can result in gross chromosomal rearrangements. In this context, bypassing c-NHEJ likely favors DSB end resection to support restart of replication forks, which could be mediated either by extensive stretches of homology, or by microhomology [18, 29, 30]. As another example, repair of DSB ends that are not readily ligated, such as those blocked by DNA or protein crosslinks [31], may also be facilitated by c-NHEJ bypass. Indeed, inhibition of c-NHEJ has been posited as a key function of some factors important for resistance to such DNA damage. For example, the requirement of the HR factor BRCA1 for cellular resistance to DNA damage caused by chemical inhibitors of poly ADP-ribose polymerase (PARP) can be partially rescued by loss of either the c-NHEJ factor Ku80, or the end resection inhibition factor 53BP1 [32, 33]. As another example, the role of Fanconi Anemia (FANC) factors for cellular resistance to DNA crosslinking agents has been shown to be suppressed by disrupting c-NHEJ in some experimental systems [34, 35], although not in others [33].

Apart from inhibiting c-NHEJ to favor end resection, other DNA damage response (DDR) factors have been implicated in distinct steps of Alt-EJ, including PARP. The influence of PARP on Alt-EJ has been examined with genetic disruption of *PARP-1*, as well as with catalytic inhibitors directed against PARP-1, which also likely act on multiple members of the PARP superfamily of proteins [36]. Specifically, inhibition of PARP causes reduced EJ frequency and radiosensitivity in Ku-deficient cells, and has been shown to reduce the frequency of DSB-induced chromosomal translocations [8, 37–39]. The role of PARP during EJ is unclear, but could include DSB end bridging and/or ligase recruitment [8, 37–39]. Apart from PARP, translesion polymerases have also been implicated in promoting insertion formation during Alt-EJ [5–8].

We have sought to identify other DDR factors that mediate Alt-EJ. Using a set of chromosomal break reporters, we screened an RNAi library targeting known DDR genes for siRNAs that cause a specific decrease in Alt-EJ, relative to an EJ event that is a combination of Alt-EJ and c-NHEJ (Distal-EJ between two tandem DSBs). From this analysis, we identified several DDR factors from diverse pathways that mediate Alt-EJ. Many of these factors also promote HR, including factors important for cellular resistance to DNA crosslinks, such as FANCA. Since bypassing c-NHEJ is important for both HR and Alt-EJ, we tested whether the role of Fanca during Alt-EJ in mouse cells is affected by c-NHEJ, and found that loss of Ku70 diminishes the influence of Fanca on Alt-EJ. In contrast, PARP-inhibition causes a reduction in Alt-EJ that is enhanced by Ku70 loss. Along these lines, we also found that the influence of the helicase/nuclease DNA2 during Alt-EJ and end resection is distinct from FANCA and PARP. Finally, we examined the effect of the cancer therapeutic Bortezomib on chromosomal break repair, since it has been identified as an inhibitor of FANC signaling [40, 41], and found that this small molecule also disrupts both HR and Alt-EJ, as well as end resection. We suggest that multiple distinct aspects of the DNA damage response are important for Alt-EJ, including FANCA mediated bypass of c-NHEJ.

## Results

### Diverse DDR factors differentially promote Alt-EJ *versus* and EJ event that is a composite of Alt-EJ and c-NHEJ

**Figure 1:**
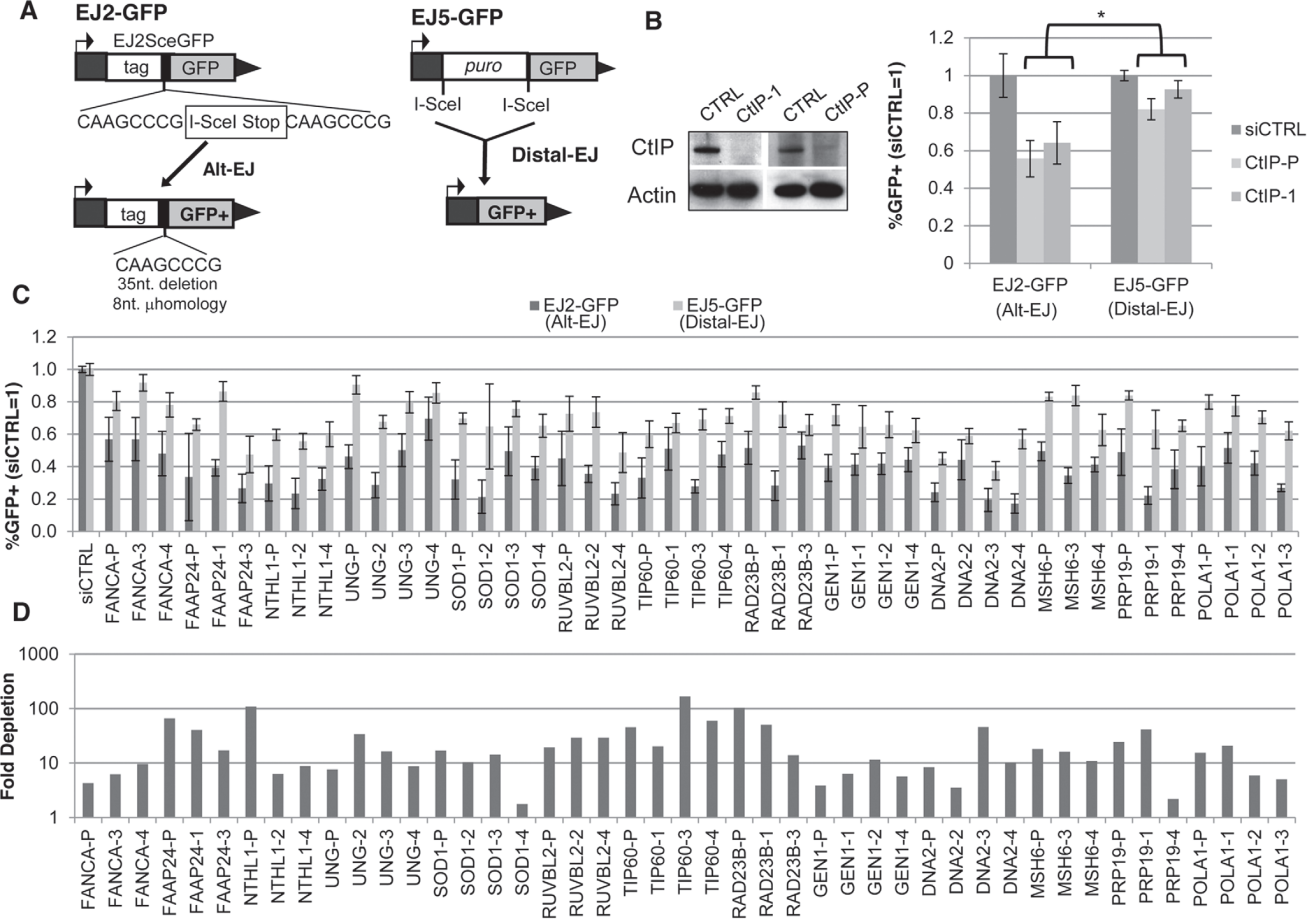
Several diverse DDR factors are differentially important for Alt-EJ relative to an EJ event that is a composite of Alt-EJ and c-NHEJ (Distal-EJ). (**A**) Shown are diagrams of the EJ2-GFP and EJ5-GFP reporters for measuring Alt-EJ and Distal-EJ, respectively. (**B**) RNAi-depletion of CtIP in U2OS cells causes a differential effect on Alt-EJ relative to Distal-EJ. Shown are immunoblot signals for CtIP and actin after transfection with a non-targeting siRNA (siCTRL), pool of four CtIP siRNAs (CtIP-P), and an individual CtIP siRNA not in the pool (CtIP-1). Shown are the frequencies of GFP+ cells for the EJ2-GFP and EJ5-GFP reporter lines after siRNA-mediated depletion of CtIP relative to parallel transfections with siCTRL, followed by transfection with an I-SceI expression vector. *P≤0.0008 distinct from the effect on Distal-EJ (*n*≥4). (**C**) Survey of DDR factors identifies 13 genes whose depletion causes a statistical difference on Alt-EJ relative to Distal-EJ. U2OS cells were transfected with siRNAs prior to EJ analysis as described in B. Shown are EJ values from 13 genes for which the siRNA pool and at least two independent siRNAs cause a significantly greater decrease in Alt-EJ *versus* Distal-EJ (*P*≤0.02, *n*≥6). (**D**) Each siRNA shown in C causes a decrease in target mRNA. Shown is the fold depletion of target mRNA after siRNA treatment, as quantified by qRT-PCR (2^ΔΔCt^, based on normalization to actin and siCTRL treated cells).

To identify mediators of Alt-EJ, we sought to determine the influence of known DDR factors on a previously described Alt-EJ reporter, EJ2-GFP [12], integrated into human osteosarcoma cells (U2OS) [42]. In this reporter, the reading frame of a GFP cassette with an N-terminal tag is disrupted by an I-SceI site followed by stop codons in all frames, which is flanked by 8 nucleotides of microhomology (Fig. 1A). Alt-EJ of an I-SceI generated chromosomal DSB that deletes the stop codons, which occurs predominantly via the use of 8 nucleotides of flanking microhomology to bridge the DSB, restores the GFP cassette and causes a 35 nucleotide deletion [12]. Notably, since this predominant Alt-EJ event shows microhomology at the repair junction, and also is enhanced by loss of c-NHEJ factors [12], the terms MMEJ and Alt-EJ are both applicable to this repair event, and hence are largely interchangeable in this context. We examined a set of DDR factors on this Alt-EJ repair event using an RNAi library targeting 238 genes with pools of 4 siRNAs per gene, by pretreating cells with each siRNA pool prior to transient transfection with the I-SceI expression vector, and subsequent analysis of the frequency of GFP+ cells using flow cytometry.

We also performed a secondary screen, since siRNAs that cause a reduced frequency of Alt-EJ using the EJ2-GFP reporter could reflect not just a reduction in this particular repair outcome, but also an overall loss of DSB repair proficiency. For this secondary screen, we examined a distinct reporter for total EJ: EJ5-GFP [12, 42]. In this reporter, a GFP cassette is separated from its promoter by a marker gene that is flanked by two tandem I-SceI sites, such that EJ repair that uses distal ends of the two I-SceI-induced DSBs restores GFP expression (Distal-EJ, Fig. 1A). These Distal-EJ events reflect a composite of c-NHEJ and Alt-EJ, because the GFP cassette can be restored by diverse EJ repair junctions (e.g. I-SceI restoration, deletions, or insertions) [12, 21, 43], and hence can occur by multiple EJ mechanisms. Consistent with this notion, GFP restoration with this reporter is not dependent on c-NHEJ factors (e.g. Ku70 or Xrcc4) [12, 21, 43]. Thus, examining both the Alt-EJ (EJ2-GFP) and the Distal-EJ (EJ5-GFP) reporters provides a means to distinguish between specific effects on Alt-EJ, *versus* relatively nonspecific effects on DSB repair that are reflected in changes to the frequency of total EJ.

Accordingly, to identify factors that specifically affect Alt-EJ, we sought to identify siRNAs that cause not only a significant decrease in the frequency of Alt-EJ (EJ2-GFP) relative to non-targeting siRNA (siCTRL), but also cause a significantly greater fold-decrease in Alt-EJ *versus* Distal-EJ (EJ5-GFP). As an example of this approach, depletion of the end resection factor CtIP has been previously shown to cause both a significant decrease in Alt-EJ, as well as a greater decrease in Alt-EJ *versus* Distal-EJ [12], which is consistent with the conclusions of other studies on CtIP and EJ [18, 44]. We confirmed this result with an individual siRNA targeting CtIP (siRNA CtIP-1) and the CtIP siRNA pool from the library (Fig. 1B, *P*≤0.0008). Thus, we included siCtIP-1 as a positive control for our screen to identify siRNA pools that cause a decrease in Alt-EJ *versus* Distal-EJ.

To perform the screen, we determined the fold change caused by each siRNA pool on the frequency of Alt-EJ and Distal-EJ (N = 2), relative to parallel siCTRL treatments. We then calculated the ratio of this fold change on Alt-EJ *versus* Distal-EJ, and performed additional repeats of several siRNA pools that appeared to cause the greatest effects on the Alt-EJ/Distal-EJ ratio. We then ranked the siRNA pools according to this Alt-EJ/Distal-EJ ratio to complete the screen (S1 and S2 Tables, S1A Fig.). Although we did identify a few genes causing a relative increase in Alt-EJ, we focused on the siRNAs showing a low Alt-EJ/Distal-EJ ratio in order to identify mediators of Alt-EJ. For this, we examined individual siRNAs for the 15 genes showing the lowest Alt-EJ/Distal-EJ ratio. As well, since one of these genes was FAAP24, which is required for recruitment of FANCA to chromatin damaged by the crosslinking agent mitomycin-C [45], and since FANCA ranked in the top 10% of the screen, we also examined individual siRNAs for FANCA. From this analysis of 16 genes, we found that 13 showed at least two individual siRNAs that caused a significantly greater decrease in Alt-EJ compared to Distal-EJ: FANCA, FAAP24, NTHL1, UNG, SOD1, RUVBL2, TIP60/KAT5, RAD23B, GEN1, DNA2, MSH6, PRP19/PSO4, and POLA1 (Fig. 1C, *P*≤0.02, Alt-EJ/Distal-EJ ratio of this data in S1B Fig.). To validate the siRNAs from these Alt-EJ mediators, we confirmed depletion of the target mRNA using quantitative RT-PCR (qRT-PCR, Fig. 1D, S1 Table).

### Many Alt-EJ mediators, including FANCA, are also important for HR

**Figure 2:**
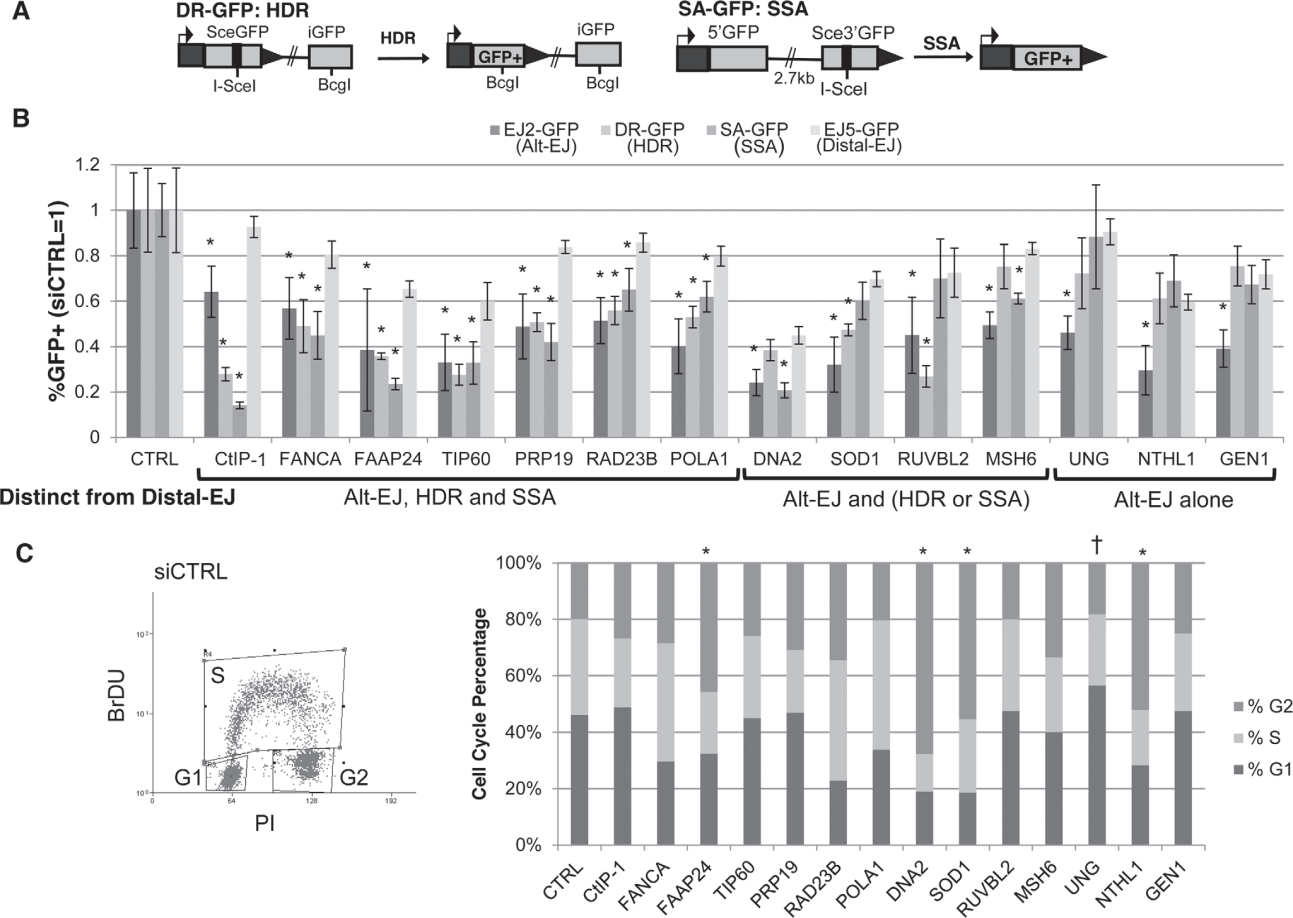
Most DDR factors identified that promote Alt-EJ also promote HR. (**A**) Shown are diagrams of the DR-GFP and SA-GFP reporters for measuring HDR and SSA, respectively. (**B**) Influence of Alt-EJ mediators identified in Fig. 1 on HDR and SSA. U2OS cells were treated with siCTRL, siRNA CtIP-1, and the siRNA pools described in Fig. 1, prior to expression of I-SceI and analysis of GFP+ cells by flow cytometry. Shown are the frequencies of GFP+ cells for Alt-EJ, HDR, SSA and Distal-EJ for each siRNA pool relative to parallel transfection with siCTRL (i.e. siCTRL = 1). Genes are clustered into three categories based on which repair events are affected differentially from Distal-EJ. *P<0.005, statistically different from the effect of the siRNA pool on Distal-EJ (*n*≥4), *P*>0.02 not considered statistically different. (**C**) Cell cycle phase distribution following siRNA treatment. U2OS cells were treated with siRNAs as in B, and subsequently labeled with BrdU, prior to staining for BrdU and PI. Shown is a representative plot from the flow cytometry analysis of siCTRL treated cells, along with the cell cycle phase percentages for each siRNA treatment. *P<0.02 distinct increase in %G2 phase, and †*P<*0.005 distinct increase in %G1 phase cells, each relative to siCTRL treated cells.

We then sought to identify commonalities among these 13 mediators of Alt-EJ by also examining their effect on HR. For this, we tested the siRNA pools in U2OS cells with reporters for HDR and SSA (Fig. 2A). DR-GFP is a reporter for HDR in that GFP expression is restored via repair that uses a homologous template (iGFP) for RAD51-dependent gene conversion [16, 46]. In contrast, SA-GFP is a reporter for SSA, in that GFP expression is restored by a repair event that uses two flanking homologous repeats to bridge the DSB, causing a deletion between the repeats [16]. For SA-GFP, GFP restoration could also occur by certain gene conversion events: long-tract gene conversion that is resolved by EJ (LTGC-EJ), or with crossing over. The relatively low frequency of these events, combined with the finding that RAD51 disruption causes an increase in GFP+ cells with this reporter, indicates that the restoration of GFP predominantly occurs via SSA [16]. However, we note that deficiencies in some DDR factors (e.g. BRCA1 and CtIP) have been shown to cause an increase in LTGC [47], such that it is important to also consider the possibility that the relative contribution of SSA *versus* LTGC-EJ could be altered by depletion of certain DDR factors. As our positive control for this analysis, we again used CtIP depletion that has been shown to cause a significant reduction in both HDR and SSA, compared to Distal-EJ [12], which we have confirmed (siRNA CtIP-1, Fig. 2B).

From this analysis, 6/13 factors showed a similar pattern to CtIP, in that RNAi of these factors caused a greater decrease in Alt-EJ, HDR, and SSA, each compared to Distal-EJ (*P*≤0.004): FANCA, FAAP24, TIP60/KAT5, PRP19/PSO4, RAD23B, POLA1 (Fig. 2B). An additional four factors showed a significant decrease in either HDR or SSA relative to Distal-EJ (*P*≤0.003): DNA2 (SSA only), SOD1 (HDR only), RUVBL2 (HDR only), and MSH6 (SSA only) (Fig. 2B). The remaining three showed a specific effect only on Alt-EJ (*P*<0.0001): UNG, NTHL1 and GEN1 (Fig. 2B).

Since Alt-EJ and HR appear more proficient in the S/G2 phases of the cell cycle [18, 48], we next considered that the above 13 Alt-EJ mediators might affect cell cycle phase distribution. In particular, we wanted to test whether depletion of these factors via siRNA causes a substantial shift to G1 phase cells, which could possibly cause a reduction in Alt-EJ and/or HR. Thus, following siRNA treatment (siCTRL, siRNA CtIP-1, and siRNA pools of the above 13 Alt-EJ mediators), we labeled S-phase cells using a pulse of bromodeoxyuridine (BrdU) incorporation, performed co-staining with the DNA dye propidium iodide (PI), and analyzed cells by flow cytometry. From this experiment, we found that siRNAs targeting a few of the genes caused a significant increase in G2 phase cells (Fig. 2C, FAAP24, DNA2, SOD1, and NTHL1, *P*<0.02), whereas only the siRNAs targeting UNG caused a significant, but modest, increase in G1 phase cells (Fig. 2C, UNG 56%, siCTRL 45%, *P*<0.005). These findings indicate that, apart from possibly UNG, the reduction in Alt-EJ and HR caused by depletion of the 13 genes described above cannot be readily attributed to an increase in G1 phase cells.

### Several other FANC factors show a similar influence as FANCA and FAAP24 on Alt-EJ and HR

**Figure 3:**
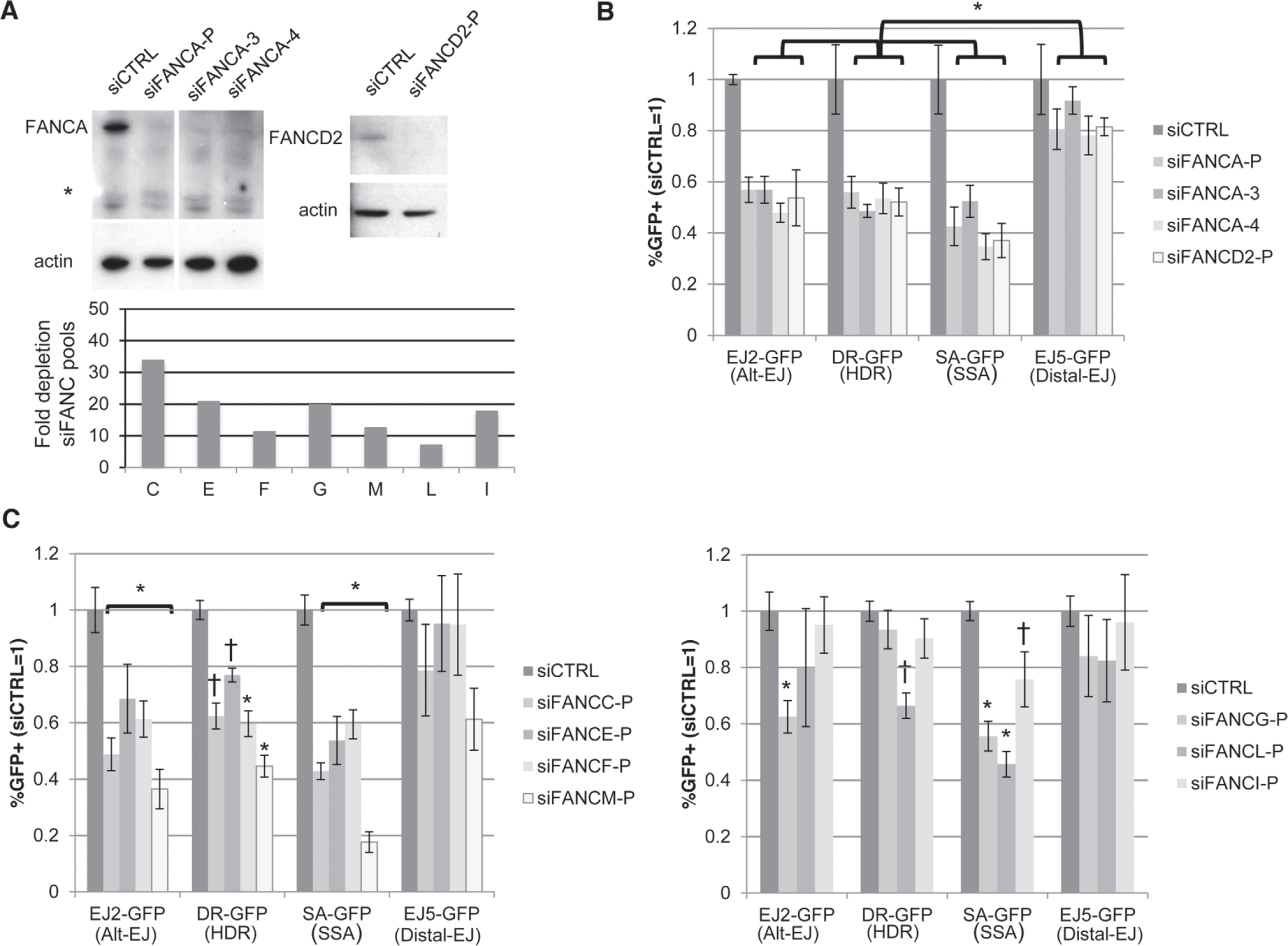
Several other FANC factors show a similar influence as FANCA on Alt-EJ and HR. (**A**) Targeting FANC genes with siRNA. Shown are immunoblot signals from U2OS cells treated with siCTRL, FANCA siRNA pool (siFANCA-P), two individual siRNAs from the siFANCA pool (siFANCA-3 and siFANCA-4), and a FANCD2 siRNA pool (siFANCD2-P). *indicates nonspecific band. Also shown is the fold decrease in target mRNA after the respective siRNA treatment, as quantified by qRT-PCR (2^ΔΔCt^, based on normalization to actin and siCTRL treated cells, as in Fig. 1D), for siRNA pools of seven FANC genes (C, E, F, G, M, L, and I). (**B**) Analysis of Alt-EJ, HDR, SSA and Distal-EJ after depletion of FANCA and FANCD2. Shown are GFP+ frequencies of U2OS cells treated with siRNAs from A and analyzed as in Fig. 2B. *P≤0.003 distinct from the effect of each siRNA on Distal-EJ (*n*≥4). (**C**) Analysis of Alt-EJ, HDR, SSA and Distal-EJ after depletion of the other FANC factors (C, E, F, G, M, L, and I). Shown are GFP+ frequencies of U2OS cells treated with siRNAs from A and analyzed as in B. *P<0.01 distinct from the effect of each siRNA on Distal-EJ (*n* = 6). †*P*<0.04 distinct from the effect of each siRNA on Distal-EJ (*n* = 6).

Since depleting both FANCA and FAAP24 caused a reduction in Alt-EJ and HR, and furthermore since we perform additional analysis below with FANCA, we considered that other FANC factors may also have a similar influence on Alt-EJ and/or HR. Namely, while siRNAs targeting other FANC genes did not cause the greatest decrease in Alt-EJ *versus* Distal-EJ relative to other genes in the library (S2 Table), we nevertheless considered the possibility that targeting these FANC genes may cause a statistical difference among different repair outcomes. For this, we examined the siRNA pools in the library that target several FANC genes, using each of the reporter assays described above. To begin with, we evaluated FANCD2, and for comparison, we included the FANCA siRNA pool and two individual FANCA siRNAs. From these experiments, we found that each of these siRNAs (siFANCA-P, siFANCA-3, siFANCA-4, siFANCD2-P) depleted the target protein as detected by immunoblot (Fig. 3A). Furthermore, each of these siRNAs showed a similar effect as the FANCA siRNA pool (siFANCA-P) on the reporter assays, in that Alt-EJ, HDR, and SSA were each reduced to a greater extent than Distal-EJ (Fig. 3B). To extend this analysis, we then examined each of the FANC core complex and associated factors represented in the library (FANCC, E, F, G, I, L, and M) [49]. We confirmed that each of these siRNA pools caused depletion of the target mRNA (S1 Table, Fig. 3A). From the reporter assay analysis, we found that siRNAs targeting FANCC, E, F, and M showed a similar pattern on repair as depletion of FAAP24, FANCA, and FANCD2 (i.e. a significant decrease in Alt-EJ, HDR, and SSA, relative to the effect on Distal-EJ, Figs 2B, 3B, 3C, *P*<0.01 for Alt-EJ and SSA, *P*<0.04 for HDR). In contrast, while siRNAs targeting FANCG, L, and I caused a significant decrease in SSA relative to Distal-EJ, only FANCG caused a relative decrease in Alt-EJ, and only FANCL caused a relative decrease in HDR (Fig. 3C, *P*<0.04). Although, the modest effects of siRNAs targeting FANCG, L, and I on repair outcome could reflect incomplete disruption of these factors, which is an inherent limitation of siRNA experiments. In any case, these results indicate that depletion of several FANC factors (C, D2, E, F, and M) show a similar pattern as depletion of FANCA and FAAP24 in causing a reduction in Alt-EJ and HR relative to Distal-EJ.

### The influence of Fanca on Alt-EJ, but not cisplatin hypersensitivity, is diminished by loss of Ku70

The above findings indicate that FANCA is important for Alt-EJ, HDR, and SSA, which are all repair events that involve bypass of c-NHEJ, in that these events are elevated in the absence of c-NHEJ factors, such as Ku70 and Xrcc4 [10, 12, 15, 21, 50]. Accordingly, these findings support the notion that the FANC pathway may be important for c-NHEJ bypass, which has been suggested by studies showing that the DNA crosslink sensitivity of FANC-deficient cells can be rescued by c-NHEJ disruption [34, 35]. However, this genetic interaction does not appear to be consistent among all experimental systems [33, 49]. Thus, to examine the genetic relationship of FANC and c-NHEJ, we sought to test how loss of c-NHEJ affects both Alt-EJ and DNA crosslinking sensitivity in FANC-deficient cells. For this, we used mouse embryonic stem (mES) cells, in which Ku is not essential for viability, in comparison to human cells that require Ku for survival, likely due to its role in telomere maintenance [51].

**Figure 4:**
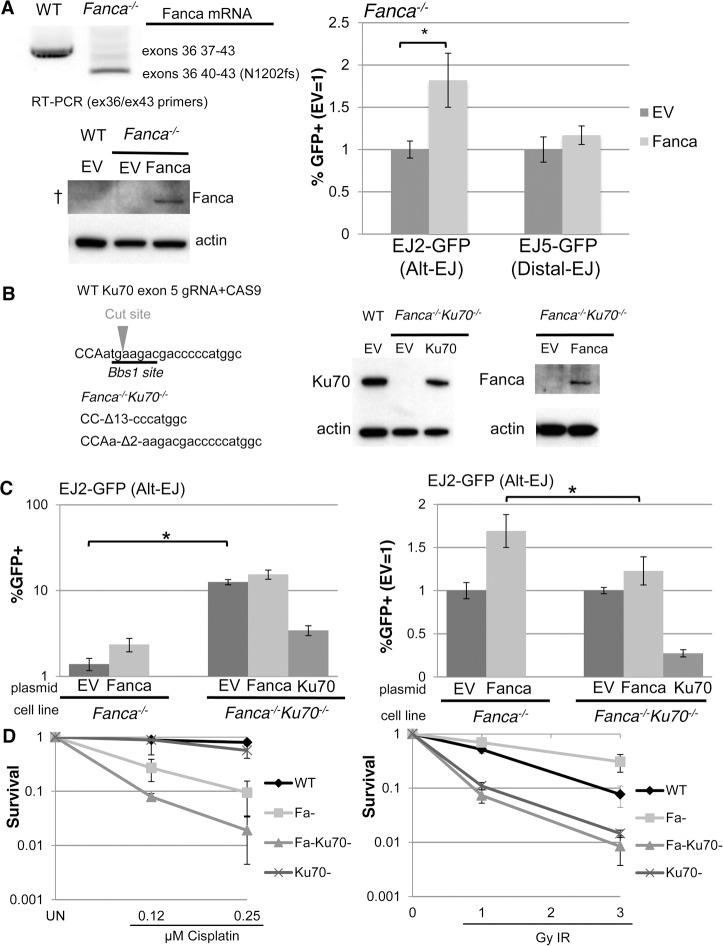
Loss of Ku70 can diminish the influence of Fanca on Alt-EJ, but not cisplatin sensitivity. (**A**) Transient expression of Fanca in *Fanca^−/−^* mES cells promotes Alt-EJ but not Distal-EJ. The EJ2-GFP and EJ5-GFP reporters were integrated into *Fanca^−/−^* mES cells. Shown are RT-PCR signals confirming the exon 37–39 deletion in *Fanca^−/−^*, which causes a frame shift at N1202. These cells were transfected with expression vectors for I-SceI along with either mouse Fanca or empty vector (EV). Repair values (%GFP+) from these transfections are shown relative to parallel EV. *P<0.0001 (*n* = 9). Also shown are immunoblot signals detecting expression of Fanca from cells transfected with the expression vector, although this anti-human FANCA antibody is unable to detect endogenous Fanca in WT (†). (**B**) Generation of *Ku70* frame-shift mutations in *Fanca^−/−^* EJ2-GFP mES cells. Shown is the predicted target cut site in *Ku70* exon 5 for a guide RNA and CAS9, along with sequence of the *Ku70* frame-shift mutations (13 and 2 nt deletions, Δ13 and Δ2, respectively) that resulted from two rounds of CAS9 expression. Shown are immunoblot signals of these *Fanca^−/−^Ku70^−/−^* mES cells for Ku70 and Fanca, following transient transfection with Ku70 or Fanca expression vectors, respectively. (**C**) Loss of Ku70 diminishes the influence of Fanca on Alt-EJ. The *Fanca^−/−^* and *Fanca^−/−^Ku70^−/−^* EJ2-GFP cell lines were co-transfected with Fanca, Ku70, or EV and I-SceI expression vectors. Shown are %GFP+ frequencies normalized to transfected cells using transfection with a GFP expression vector in parallel. *P<0.0001 (*n* = 11). On the right are the fold changes in GFP+ frequencies relative to EV. *P<0.0001 (*n* = 11). (**D**) Loss of *Ku70* causes cisplatin and IR hypersensitivity in *Fanca-/-* cells. WT, *Fanca^−/−^*, *Fanca^−/−^Ku70^−/−^*, and *Ku70^−/−^* mES cell lines were treated continually with cisplatin, or exposed to IR (Cs137), and cultured to allow colony formation. Shown is fraction clonogenic survival relative to untreated cells (UN).

Specifically, we examined the influence of Fanca on Alt-EJ and DNA crosslink sensitivity in mES cells, both in the presence and absence of Ku70. For this, we integrated the EJ2-GFP and EJ5-GFP reporters into a previously described *Fanca^−/−^* mES cell line [51–53], which we confirmed involves deletion of exons 37 to 39, causing a frame-shift at position N1202 (Fig. 4A). Then, we transfected these cell lines with an expression vector for I-SceI, along with either an expression vector for Fanca, or the associated empty vector (EV). By immunoblotting with an anti-human FANCA antibody, we detected Fanca in cells transfected with the Fanca expression vector, although this antibody was unable to detect endogenous Fanca in WT cells (Fig. 4A). In comparison to parallel EV transfections of *Fanca^−/−^* cells, we found that expression of Fanca caused a significant increase in Alt-EJ (1.8 fold, *P*<0.0001), but not Distal-EJ (Fig. 4A), which is consistent with our above findings that FANCA promotes Alt-EJ in U2OS cells. To examine the effect of Ku70 on Alt-EJ in *Fanca^−/−^* cells we introduced frame-shift mutations in both alleles of *Ku70* in the *Fanca^−/−^* EJ2-GFP cells using CAS9-mediated genome engineering [54], which we confirmed caused a loss of the Ku70 protein (Fig. 4B). With these cells, we expressed I-SceI, along with either a complementation vector for Fanca, Ku70, or EV (expression confirmed by immunoblotting, Fig. 4B). From this analysis, we found that loss of Ku70 in *Fanca^−/−^* cells caused an increase in Alt-EJ (Fig. 4C, 9-fold, *P*<0.0001), which was substantially reduced with transient expression of Ku70 (Fig. 4C, 3.7-fold, *P*<0.0001). The finding that Alt-EJ in *Fanca^−/−^Ku70^−/−^* cells was not completely reduced to the level of *Fanca^−/−^* cells by transient expression of Ku70 likely reflects limitations of this approach to precisely mimic endogenous expression, which is consistent with prior findings with transient Ku70 expression [16]. Using this pair of *Fanca^−/−^Ku70^−/−^* and *Fanca^−/−^* cells, we next examined the effect of transient Fanca expression on Alt-EJ. From these experiments, we found that the fold effect of Fanca expression on Alt-EJ is significantly reduced in *Fanca^−/−^Ku70^−/−^* cells, compared to *Fanca^−/−^* cells (Fig. 4C, 1.2-fold and 1.7 fold, respectively, *P*<0.0001). These results indicate that loss of Ku70 can diminish the influence of Fanca on Alt-EJ.

To further examine the genetic interaction between *Fanca* and *Ku70* in response to DNA damage, we tested the effect of clastogen exposure on clonogenic survivial of the above described mES cell lines, as well as a previously described *Ku70^−/−^* cell line [55], and a WT line. Specifically, we exposed these cells to different concentrations of the DNA crosslinking agent cisplatin, or doses of ionizing radiation (IR), and compared colony formation to untreated cells (Fig. 4D). From this analysis, we found that *Fanca^−/−^* cells show substantial hypersensitivity to cisplatin, as compared to both WT and *Ku70^−/−^* (Fig. 4D, *P*≤0.006). Furthermore, while *Ku70^−/−^* cells were not obviously more sensitive to cisplatin than WT, the *Fanca^−/−^Ku70^−/−^* cells showed even greater cisplatin sensitivity than the *Fanca^−/−^*cells (Fig. 4D, *P*≤0.01). This latter finding is consistent with a previous study of *Fancd2^−/−^* and *Fancd2^−/−^Ku80^−/−^* mouse cells [33], but not in experiments from other systems [34, 35, 49]. In contrast to cisplatin response, both *Ku70^−/−^* and *Fanca^−/−^Ku70^−/−^* showed IR hypersensitivity, compared to both WT and *Fanca^−/−^* cells (Fig. 4D). As well, *Fanca^−/−^* cells did not show hypersensitivity to IR, and indeed were modestly IR resistant compared to WT (Fig. 4D, *P*<0.0001 at 3 Gy IR). Thus, *Ku70* appears important for IR resistance regardless of *Fanca*, but is important for cisplatin resistance specifically in *Fanca^−/−^* cells. These findings indicate that loss of Ku70 can diminish the role of Fanca in promoting Alt-EJ, but not DNA crosslink resistance.

### Effect of PARP inhibition on Alt-EJ is enhanced by loss of Ku, and hence is distinct from loss of FANCA

**Figure 5:**
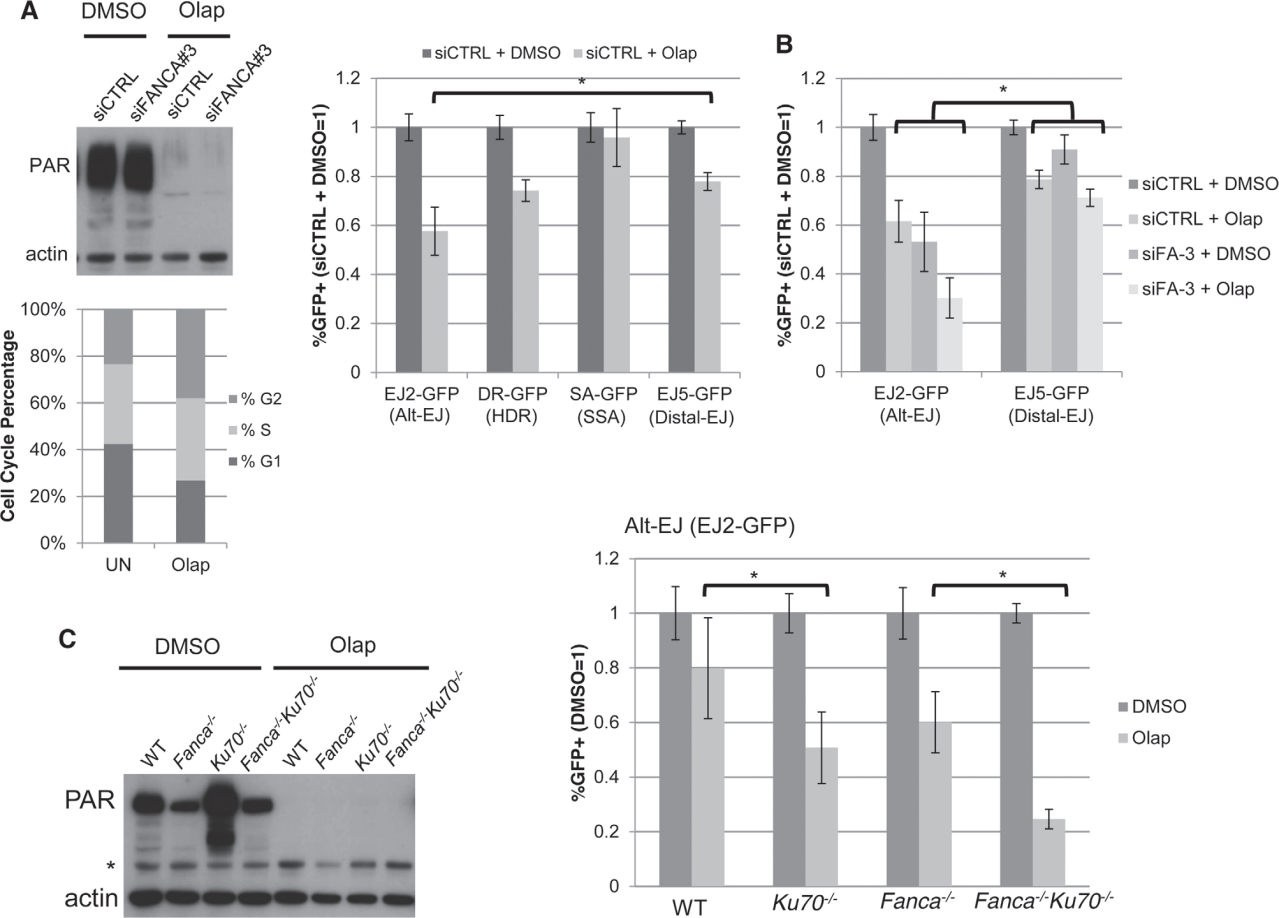
PARP inhibition causes a decrease in Alt-EJ that is enhanced by Ku70 loss. (**A**) PARPi treatment (Olaparib) causes a specific decrease in Alt-EJ, compared to Distal EJ. Shown are the immunoblot signals for PARylated protein and actin after treatment of U2OS cells with 5 μM of the PARPi Olaparib (2 days). Also shown is the cell cycle profile of siCTRL treated U2OS cells incubated with 5μM Olaparib (Olap), or mock treated (UN) prior to BrdU labeling and PI staining as in Fig. 2C. The U2OS reporter cell lines described above were transfected with the I-SceI expression vector and treated with 5 μM Olaparib (Olap) or vehicle (DMSO). Shown are the %GFP+ cells for each reporter relative to vehicle treatment (DMSO). *P<0.0001 distinct from the effect on Distal-EJ (*n* = 13). (**B**) Effect of PARPi on Alt-EJ is approximately additive with loss of FANCA. Shown are GFP+ frequencies for U2OS cells treated with an siRNA targeting FANCA (siFANCA-3) or siCTRL, and subsequently examined as in A, relative to siCTRL/DMSO treated cells. *P<0.0001 distinct from the effect on Distal-EJ (n≥8). (**C**) Loss of Ku70 enhances the effect of PARPi on Alt-EJ in mES cells. Shown are immunoblot signals for PARylated protein and actin for WT, *Fanca^−/−^*, *Ku70^−/−^* and *Fanca^−/−^Ku70^−/−^* mES cells treated with Olaparib (2 μM, 2 days) or vehicle (DMSO). *indicates nonspecific band. Each of these mES cells with the EJ2-GFP reporter were transfected with I-SceI and treated with 2 μM Olaparib (Olap) or vehicle (DMSO). Shown is the frequency of %GFP+ cells relative to vehicle treated (DMSO). *P≤0.001 (*n*≥9).

In contrast to the above findings with FANCA, PARP function during DSB repair has been suggested to be independent of c-NHEJ. Namely, chemical PARP inhibitors cause radiosensitivity and reduced EJ frequency, in a manner enhanced by Ku-deficiency [8, 37–39]. Thus, we considered whether PARP inhibition might have a distinct influence on DSB repair *versus* FANCA. We first examined the effect of the PARP inhibitor (PARPi) Olaparib, on each of the U2OS reporter cell lines described above (Figs 1A and 2A). Inhibition of PARP activity via Olaparib was confirmed by immunoblotting analysis of PARylated protein (Fig. 5A). Furthermore, we found that Olaparib treatment did not obviously affect cell cycle phase distribution (Fig. 5A). From the reporter assay analysis, we found that Olaparib treatment caused a significantly greater decrease in Alt-EJ compared to Distal-EJ (Fig. 5A, 1.7 fold and 1.3 fold, respectively, *P*<0.0001). In contrast, the effect of Olaparib on HDR was the same as Distal-EJ, and SSA was affected to a lesser degree than either (Fig. 5A). Thus, in contrast to FANCA and many other DDR factors that cause a similar effect on Alt-EJ and HR, the PARPi Olaparib appears to cause a relatively specific decrease in Alt-EJ.

This finding raised the possibility that the influence of FANCA *versus* Olaparib on Alt-EJ may be distinct, which we first tested co-treatment experiments with Olaparib and siFANCA in U2OS cells. From these experiments, we found that Olaparib and siFANCA each caused a significant decrease in Alt-EJ compared to Distal-EJ as shown above, and that the effect of combined treatment was approximately additive (Fig. 5B). We then tested whether the effect of Olaparib on Alt-EJ might be affected by Ku70 loss, using the mES cell lines described above. As with the U2OS experiments, inhibition of PARP activity in mES cells with Olaparib was confirmed by immunoblot analysis of PARylated protein (Fig. 5C). We found that Olaparib treatment caused a greater decrease on Alt-EJ in *Ku70^−/−^* cells compared to WT (Fig. 5C, 1.6-fold, *P*<0.001), and in *Fanca^−/−^Ku70^−/−^* cells compared to *Fanca^−/−^* cells (Fig. 5C, 2.4-fold, *P*<0.0001). Olaparib also appeared to cause a modestly greater decrease on Alt-EJ in *Fanca^−/−^* cells, compared to WT, although this effect was not statistically significant. In any case, these results indicate that the effect of PARP inhibition on Alt-EJ is enhanced by Ku70 loss, whereas the effect of Fanca disruption on Alt-EJ is diminished by Ku70 loss (Fig. 4). Accordingly, we suggest that Fanca disruption and Olaparib treatment have distinct effects on Alt-EJ.

### The influence of the helicase/nuclease DNA2 on Alt-EJ and end resection is distinct from FANCA and PARP inhibition

**Figure 6:**
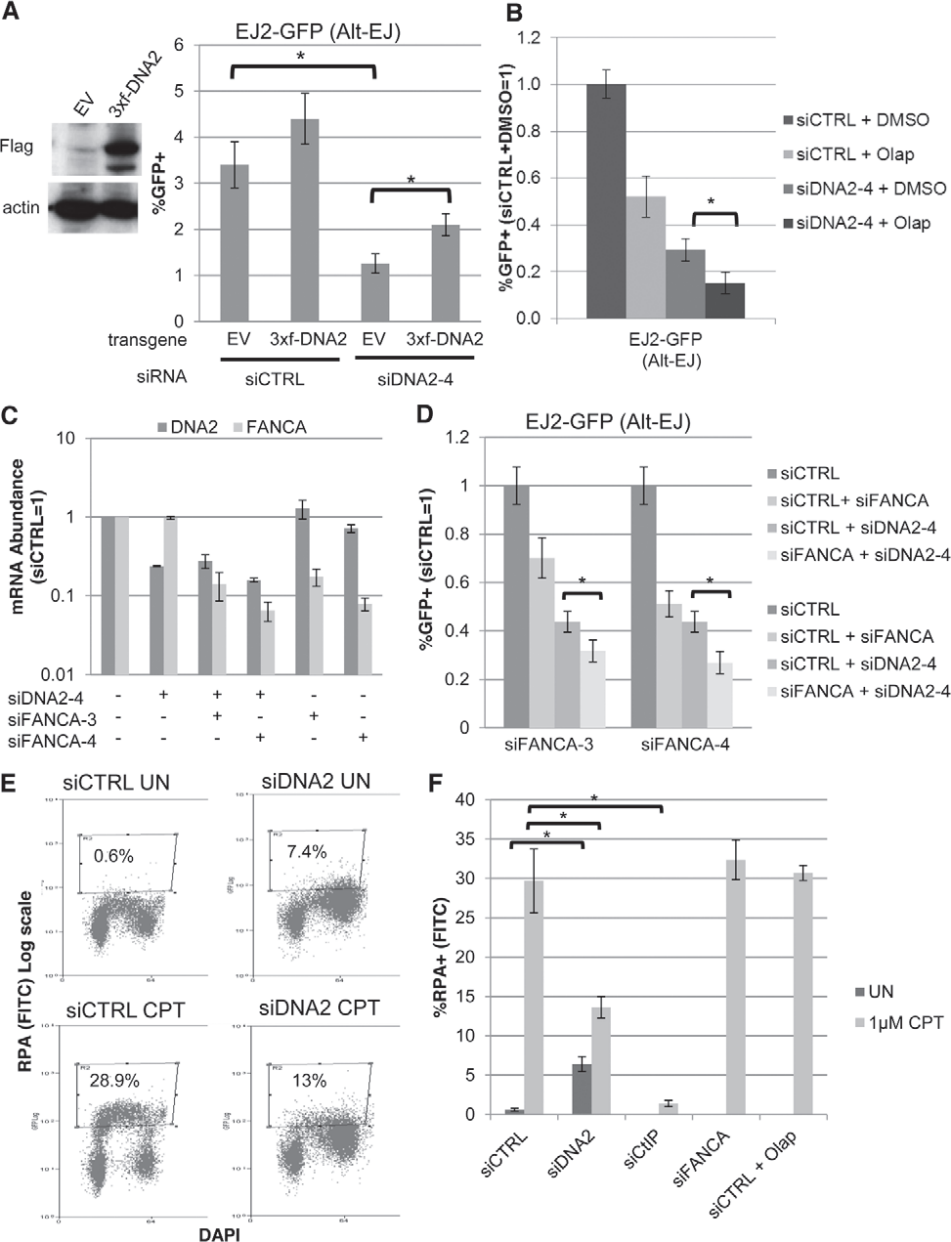
DNA2 has distinct effects on Alt-EJ and end resection, compared to FANCA and PARP. (**A**) Stable expression of DNA2 increases Alt-EJ in cells treated with an individual siRNA targeting endogenous DNA2 (siDNA2–4). U2OS EJ2-GFP cells were transduced with an expression cassette for DNA2 with a 3xFlag immunotag and silent mutations for resistance to siDNA2–4, or transduced with EV. These cell lines were treated with siDNA2–4 or siCTRL, and transfected with expression vectors for I-SceI, or a GFP expression vector in parallel to quantify percent transfected cells. Shown are the %GFP+ frequencies from the I-SceI transfections, normalized to transfected cells. *P<0.0001 (*n* = 9). (**B)** PARPi treatment and DNA2 depletion cause an approximately additive decrease in Alt-EJ. U2OS EJ2-GFP cells were treated with siCTRL or siDNA2–4 prior to expression of I-SceI and treatment with 5 μM Olaparib (Olap) or vehicle (DMSO). Shown are %GFP+ frequencies relative to siCTRL/DMSO treated cells. *P<0.0001 (*n*≥6). (**C**) The relative mRNA abundance of FANCA and DNA2 is shown for U2OS cells treated with siCTRL, siDNA2–4, siFANCA-3, and/or siFANCA-4 (qRT-PCR analysis as in Fig. 1D, fold reduction based on 2^ΔΔCt^). (**D**) RNAi targeting of DNA2 and FANCA cause an approximately additive decrease in Alt-EJ. Shown are the GFP+ frequencies of EJ2-GFP U2OS cells treated with siDNA2–4 alone or in combination with siFANCA-3 or siFANCA-4, prior to examining Alt-EJ as in B, each normalized to siCTRL. *P<0.0001 (*n*≥6). (**E**) End resection assay. Cells were detergent extracted prior to fixation and staining with RPA and DAPI, which was analyzed for individual cells by flow cytometry. Shown are representative plots for siRNA-treated (siCTRL or siDNA2 pool) U2OS cells incubated with camptothecin (CPT) or mock treated (UN). (**F**) RNAi targeting of DNA2 and CtIP, but not FANCA, nor treatment with Olaparib, causes a decrease in end resection. Cells were treated with siRNA and Olaparib prior to treatment with CPT for the end resection assay. Shown is the percentage of U2OS cells with chromatin-bound (i.e. detergent-resistant) RPA staining for the given siRNA and drug treatments, as quantified using the assay shown in E. *P<0.0002 (*n*≥3).

Among the genes besides FANCA that we identified as mediating Alt-EJ, we sought to further examine DNA2, since the siRNA pool targeting DNA2 caused the greatest decrease in Alt-EJ/Distal-EJ ratio in the screen (S2 Table), and because this factor has been implicated in end resection [56]. Furthermore, DNA2 has been shown to co-immunoprecipitate with FANCD2 [57], and appears to influence the requirement for the FANC pathway in the response to DNA crosslinking agents [58]. To begin with, we examined the effect of expressing DNA2 on Alt-EJ in U2OS cells, using stable integration of an expression cassette for 3xFlag-tagged DNA2 with silent mutations for resistance to an individual DNA2 siRNA (siDNA2–4), which we compared to cells transduced with an EV (Fig. 6A, expression confirmed by Flag immunoblot). We then treated these cells with siDNA2–4 or siCTRL, and examined the frequency of Alt-EJ after I-SceI expression. In cells treated with siDNA2–4, we found that those expressing 3xFlag-DNA2 showed a significantly greater frequency of Alt-EJ compared to EV cells (Fig. 6A, 2-fold, *P*<0.0001). While Alt-EJ was not restored to siCTRL levels by 3xFlag-DNA2 expression, this finding is consistent with a previous report showing partial complementation of DNA2 function during genome maintenance using a similar experimental system [59]. We then tested whether the influence of DNA2 on Alt-EJ is distinct from that of PARPi and/or FANCA, using co-treatment experiments in the U2OS reporter cells. From these experiments, we found that siDNA2–4 treatment caused an approximately additive decrease in Alt-EJ with Olaparib treatment (Fig. 6B), as well as with siFANCA-3 or siFANCA-4 treatment (co-depletion of DNA2 and FANCA mRNA confirmed by RT-PCR, Fig. 6C, D).

The above findings that depletion of DNA2 causes an approximately additive decrease in Alt-EJ with either FANCA depletion or Olaparib treatment indicates that the influence of DNA2 on Alt-EJ may be distinct from FANCA or PARP inhibition. Accordingly, we sought to examine whether these factors may also have distinct effects on other aspects of the DNA damage response, specifically end resection. Namely, we considered that depletion of DNA2, but not FANCA or PARP inhibition, may cause a decrease in end resection. We base this hypothesis on findings in *S. cerevisiae*, as well as mammalian cells, that DNA2 mediates end resection [56, 57, 60]. To test this, we employed a recently described flow cytometry-based assay for end resection, which uses a ssDNA binding protein (RPA, specifically RPA32) as a marker of ssDNA formation [61]. Specifically, this assay measures chromatin-bound (i.e. detergent-resistant) ssDNA binding protein (RPA32), which is detected by RPA32 immunostaining, and combined with counterstaining with the DNA dye DAPI. This assay is designed to measure end resection at DNA replication forks that are blocked by the topoisomerase I poison camptothecin (CPT), and such end resection was shown to be dependent on CtIP [61]. A limitation of this assay is that end resection at blocked replication forks induced by CPT treatment is not necessarily equivalent to end resection at DSBs. Nevertheless, this assay provides a quantitative measure of end resection. To begin with, we confirmed previous findings that CPT treatment of U2OS cells significantly induced a population of cells with chromatin-bound RPA staining, which was abolished in cells treated with an siRNA targeting CtIP (Fig. 6E, F, S2A, B Fig.). We next examined cells treated with siRNAs targeting DNA2, as well as each of the other 13 Alt-EJ mediators (siRNA pools), and cells treated with Olaparib. We found that DNA2 depleted cells showed a shift towards G2 phase cells, which was consistent with the cell cycle analysis described above (Fig. 2C). Without CPT treatment, these G2 phase cells showed a significant increase in RPA signal, compared to siCTRL treated cells without CPT treatment (Fig. 6E, F, *P*<0.0001). Such RPA staining may reflect defects in processing replication intermediates in DNA2 deficient cells [59, 62]. After CPT treatment, the frequency of cells with RPA signal was significantly lower for DNA2 depleted cells compared to siCTRL treated cells (6E, F, *P*<0.0002), although they were not completely abolished as with CtIP depletion. In contrast, cells that were depleted of the other 12 Alt-EJ mediators we described above (including FANCA), or cells treated with Olaparib, did not show an obvious decrease in the frequency of cells with CPT-induced RPA signal, compared to siCTRL treated cells (Fig. 6E, F, S2B Fig.). These findings indicate that DNA2 depletion, but not FANCA depletion or PARP-inhibtion, causes a reduction in end resection by this assay. These findings support the notion that DNA2 has a distinct influence from FANCA and PARP-inhibition during Alt-EJ and end resection.

### Bortezomib inhibits not only FANC signaling, but also HR, Alt-EJ, and end resection

**Figure 7:**
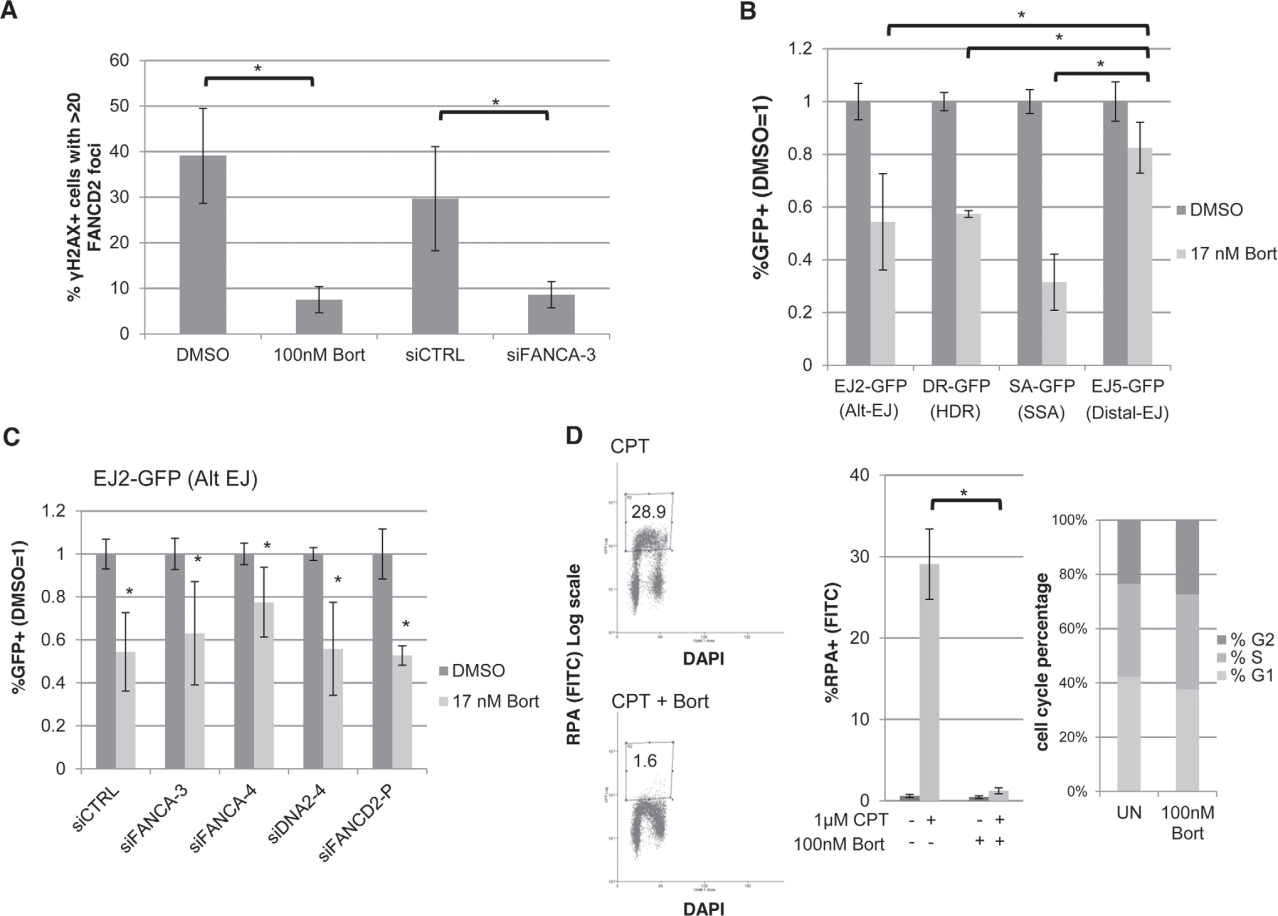
The proteasome inhibitor Bortezomib inhibits not only FANC signaling, but also HR, Alt-EJ, and end resection. (**A**) Bortezomib treatment disrupts FANCD2 IRIF. U2OS cells were treated with Bortezomib (100 nM, 4 hr before IR treatment) or vehicle (DMSO) as well as with an individual FANCA siRNA (siFANCA-3) or non-targeting siRNA (siCTRL). Subsequently, cells were treated with 10 Gy IR and allowed to recover (4 hr) prior to fixation and immunostaining for FANCD2 and the DSB marker γH2AX. Cells positive for γH2AX foci were scored for FANCD2 foci. Shown is the percentage of U2OS cells with >20 FANCD2 foci for each treatment. *P≤0.01 (*n*≥3). (**B**) Bortezomib treatment causes a greater reduction in Alt-EJ, HDR, and SSA, relative to Distal-EJ. The U2OS reporter cell lines were treated with siCTRL prior to transfection with expression vectors for I-SceI, or a GFP expression vector in parallel to quantify percent transfected cells, and treated with Bortezomib (17 nM) or vehicle (DMSO). The concentration of Bortezomib in these experiments was lower than that used in A, since the reporter assays require a dose that is sublethal for several days of continual treatment. Shown are %GFP+ frequencies normalized to transfected cells, and compared to vehicle (DMSO) treated. *P≤0.004 distinct from the effect on Distal-EJ (*n*≥5). (**C**) Bortezomib treatment inhibits Alt-EJ independently of FANCA, FANCD2 and DNA2. The EJ2-GFP reporter assay was performed as in B for cells pre-treated with siRNAs targeting FANCA, DNA2 and FANCD2. Shown are the GFP+ frequencies normalized as in B. *P≤0.009 distinct from vehicle (DMSO) treated (*n*≥6). (**D**) Bortezomib treatment causes a marked decrease in end resection but does not affect cell cycle phase distribution. U2OS cells were treated with Bortezomib as in A prior to end resection analysis as in Fig. 6E. Shown are representative flow cytometry plots for the end resection assay, and the percentage of cells with chromatin-bound RPA staining quantified using this assay. *P<0.0001 (*n*≥3). Also shown are cell cycle phase percentages following the same Bortezomib treatment as for the end resection assay, based on BrdU labeling and PI staining as in Fig. 2C.

Since our above analysis indicates that disrupting the FANC signaling pathway can cause a reduction in both HR and Alt-EJ, we sought to test whether a small molecule inhibitor of this pathway could have a similar effect on repair. For this, we examined the small molecule 26S proteasome inhibitor Bortezomib [40], which has been shown to disrupt FANC signaling, inhibit HDR, and cause DNA crosslink sensitization [41, 63, 64]. Indeed, Bortezomib has been proposed as a possible therapeutic crosslink sensitizer for FANC proficient tumors [41, 63, 64], particularly since it is already used as a cancer therapeutic for multiple myeloma [40]. Thus, given these prior studies demonstrating Bortezomib as an inhibitor of the FANC pathway, and because of the clinical relevance of this small molecule as a cancer therapeutic, we sought to examine the influence of Bortezomib on the DDR and DSB repair outcomes. First, we tested whether Bortezomib treatment affects FANC signaling by examining FANCD2 accumulation into ionizing radiation induced foci (IRIF, DSB induction confirmed by γH2AX staining, S3 Fig.). We found that Bortezomib treatment caused a marked reduction in the frequency of FANCD2 IRIF, which is similar to the effects of FANCA depletion (siFANCA-3) (Fig. 7A, 5-fold and 3.5-fold respectively), and is consistent with previous studies [41]. Next, we tested the effect of Bortezomib treatment on the DSB reporter assays in U2OS cells, using a concentration that was sublethal over the course of the experiment (17 nM). From this experiment, we found that Bortezomib treatment caused a significantly greater decrease in Alt-EJ, HDR, and SSA, compared to Distal-EJ (Fig. 7B, *P*<0.004). Thus, Bortezomib treatment causes a similar pattern on DSB repair as depletion of FANCA and several other FANC factors (Fig. 3).

However, since Bortezomib inhibits the FANC pathway as a proteasome inhibitor [41], we considered that the effects of Bortezomib on Alt-EJ may not necessarily be dependent on the FANC pathway, and furthermore that Bortezomib may disrupt other parts of the DDR. To begin with, we examined the effect of Bortezomib treatment on Alt-EJ in cells that had been treated with siRNAs targeting FANCA, DNA2 and FANCD2, and found Bortezomib treatment caused a reduction in Alt-EJ in each of these instances (Fig. 7C). Thus, the reduction in Alt-EJ via Bortezomib treatment is not dependent on the FANC pathway. Accordingly, we next examined whether Bortezomib treatment may affect other aspects of the DDR. For this, we treated cells with Bortezomib with the same conditions as the FANCD2 IRIF experiment (Fig. 7A), and examined cell cycle distribution and end resection, using the flow cytometry assays described above (Figs 2C, 6E, respectively). From this analysis, we found that Bortezomib treatment did not obviously affect cell cycle phase distribution or BrdU incorporation, but caused a striking loss of end resection (Fig. 7D, cells with RPA staining after co-treatment of CPT and Bortezomib was substantially reduced compared to CPT treatment alone, *P*<0.0001). Thus, Bortezomib treatment not only disrupts FANC signaling, but also inhibits end resection, HR, and Alt-EJ.

## Discussion

We have sought to characterize factors that influence Alt-EJ, since many cancer-associated chromosomal rearrangements show hallmarks of Alt-EJ (e.g. microhomology) [2–4], and since this DSB repair pathway likely affects cellular responses to clastogenic therapeutics. From a targeted RNAi screen of known DDR factors, we identified 13 genes that mediate Alt-EJ, and found that the majority of these also mediate HR (HDR and/or SSA), as shown previously for the end resection factor CtIP [12, 18]. While some of these factors had not been clearly identified as HR mediators (FAAP24, RAD23B, POLA1, SOD1), the other factors have been shown to influence HR. The TIP60 chromatin remodeling complex, which includes TIP60/KAT5 and RUVBL2, has been shown to promote HDR [65], and may function to acetylate nucleosomes proximal to DSBs to block recruitment of 53BP1 [66], which is a factor that inhibits HDR, SSA, and Alt-EJ [25–28]. The nuclease/helicase DNA2 is a major end resection factor in *S. cerevisae* [56], and has been shown to promote HR and end resection in mammalian cells [57, 60], which we have confirmed here. The influence of the mismatch repair factor MSH6 on HR is somewhat complex, as mismatch repair has both pro and anti-HR activities [21, 67], although our finding here that MSH6 promotes SSA is consistent with findings in *S. cerevisae* [68]. Factors important for the FANC pathway, including FANCA, have been shown to promote HDR and SSA [52, 69]. Finally, the ubiquitin ligase PRP19/PSO4 has been shown in a recent study to promote HDR [70].

This finding that Alt-EJ and HR share several common mediators supports a model whereby Alt-EJ may often be important for completing repair of aborted HR initiation events. Namely, DDR factors that mediate both HR and Alt-EJ may promote initiation of HR under circumstances where HR is not readily finished (i.e. absence of the sister chromatid), which would then rely on Alt-EJ to guard against chromosome loss. An important initiation step of HR or Alt-EJ is likely bypass of c-NHEJ, since c-NHEJ-deficient cells (e.g. Ku70 and XRCC4 deficient) show substantially elevated levels of HDR, SSA, and Alt-EJ [9–13]. Accordingly, initiation of HR or Alt-EJ may be important under conditions whereby c-NHEJ is not feasible, such as DSB ends that are blocked by DNA crosslinks [31] or base damage, or for one-ended DSBs that arise during DNA replication. Consistent with this model, we have found that numerous factors important for DNA crosslink resistance (i.e. FAAP24, several FANC factors, and PRP19/PSO4) [49, 71, 72], appear to mediate HDR, SSA, and Alt-EJ.

Along these lines, a role for the FANC pathway in bypassing c-NHEJ was suggested by studies showing that the DNA crosslink sensitivity of FANC-deficient cells can be rescued by loss of c-NHEJ. Specifically, such suppression of DNA crosslink hypersensitivity was shown for FANCC-deficient chicken DT40 cells with deletion of Ku70 [34], Fancd2-deficient *C. elegans* with loss of Ligase IV, and FANCA or FANCD2-deficient human cells with disruption of DNA-PKcs [35]. Additional evidence that FANCA may influence EJ includes reduced DNA ligation in cell-free extracts from FANCA-deficient human cells [73], as well as altered class switch recombination junctions (greater intra-switch recombination) in *Fanca^−/−^* mice [74]. Our findings support the notion that the FANC pathway may inhibit c-NHEJ, in that FANCA promotes several repair events that are enhanced by loss of c-NHEJ (Alt-EJ, SSA, and HDR), and that the influence of Fanca on Alt-EJ in mES cells is diminished by loss of Ku70. The mechanism of such c-NHEJ inhibition is unclear, which in part reflects our relatively limited understanding of how c-NHEJ factors may be blocked from associating with DSBs and/or evicted from DSBs, the latter of which may include targeted degradation of c-NHEJ factors [75]. Distinct from these findings with Alt-EJ, we found that loss of Ku70 did not rescue DNA crosslink (cisplatin) sensitivity of Fanca-deficient cells, and indeed caused hypersensitivity, which is consistent with previous findings with *Fancd2^−/−^Ku80^−/−^* mouse cells [33]. Apart from differences in the specific FANC and c-NHEJ-deficiencies tested in different studies, the distinct cellular toxicity to DNA crosslinking agents might also reflect the diverse mechanisms of cell death caused by such clastogens (e.g. apoptosis, necrosis, and mitotic catastrophe) [76], which could be affected by cell type and experimental conditions. In summary, we suggest that the FANC pathway plays a role in bypass of c-NHEJ that is important for DSB repair and cellular response to DNA crosslinks, and hence this function could be a therapeutic target for tumor sensitization to these clastogens. However, the FANC pathway is likely important for additional aspects of the cellular response to DNA crosslinks, such that loss of the c-NHEJ pathway is not sufficient in all systems to rescue DNA crosslinking hypersensitivity.

Apart from inhibition of c-NHEJ per se, the initiation of end resection is also likely an important step of Alt-EJ, since we found that the factors CtIP and DNA2 appear to mediate Alt-EJ, as well as end resection at replication forks blocked by CPT. Although, the influence of DNA2 on this assay is distinct from CtIP, in that CPT-induced RPA staining was abolished with CtIP depletion, but not DNA2 depletion. Furthermore, DNA2 depleted cells without CPT treatment showed elevated levels of chromatin-bound RPA in G2 phase cells, as compared to siCTRL treated cells, which may reflect ssDNA caused by defects in processing DNA replication intermediates [59, 62]. Along these lines, the end resection associated with blocked DNA replication that is measured by this assay (i.e. chromatin-bound RPA induced by CPT) may not necessarily be equivalent to end resection that mediates Alt-EJ, or even HR of DSBs. For instance, relatively extensive end resection may be required to generate substantial RPA staining, but such end resection may not be required for Alt-EJ. Furthermore, c-NHEJ bypass to facilitate Alt-EJ or HR of DSBs may not be rate limiting for end resection at replication forks that are blocked by CPT. Nevertheless, the role of DNA2 during Alt-EJ and end resection appears distinct from that of FANCA, since FANCA depletion did not obviously cause a loss of end resection, and furthermore since depletion of FANCA and DNA2 caused an approximately additive decrease in Alt-EJ.

Similar to FANCA, the remaining Alt-EJ mediators that we examined did not cause an obvious effect on end resection, at least by the assay system employed in our study, which indicates that some of these factors may play distinct roles during Alt-EJ. Certainly, it is conceivable that such factors are important for bypass of c-NHEJ, as we have suggested for FANCA, but of course they could also influence other aspects of this pathway that is currently poorly understood, such as short-range 5’ DSB end resection that does not cause RPA recruitment, annealing of microhomology, DNA synthesis that is primed by the annealed microhomology, ssDNA tail processing, and/or ligation. For example *POLA1*, the catalytic subunit of Pol α, could conceivably initiate microhomology-mediated DNA synthesis to facilitate DSB end synapsis, which is consistent with the function of Pol α to synthesize short DNA primers during initiation of DNA replication [77]. In related findings from *S. cerevisiae*, repair synthesis primed by microhomology has been proposed to be mediated by another replicative polymerase (Pol δ), based on findings with the non-essential subunit *POL32* [78]. Considering another step of Alt-EJ, the GEN1 nuclease could conceivably promote short-range 5’ end resection, based on its 5’ flap cleavage activity [79, 80]. Additionally, we found that Alt-EJ is mediated by the UNG and NTHL1 DNA glycosylases [81, 82]. The role of these particular factors during Alt-EJ is unclear, although notably UNG is also important for a programmed EJ event, class switch recombination, which may involve non-catalytic functions of UNG [81]. Another possible role for DNA glycosylases during Alt-EJ could be cleavage of nucleotide base damage near DSBs, since such processed DSB ends may be more prone to Alt-EJ.

In addition to these factors, we also find that inhibiting PARP activity causes a specific decrease in Alt-EJ relative to HR, indicating that PARP inhibition does not affect all repair events that are inhibited by c-NHEJ. Indeed, we found that the effect of PARPi on Alt-EJ is enhanced by loss of c-NHEJ (e.g. Ku70-deficiency), which is consistent with other reports showing that PARPi causes radiosensitization and end-joining defects that are amplified by Ku-deficiency [37–39]. Furthermore, PARPi effects on Alt-EJ were approximately additive with loss of DNA2 and FANCA, indicating that PARPi affects a distinct step during repair. These findings support a c-NHEJ-independent influence of PARPi on Alt-EJ, which could include direct effects on end bridging and/or ligation [8, 37–39]. With respect to mechanism, we note that PARPi treatment is not equivalent to genetic loss of *PARP-1* and *PARP-2*, and may function to block repair by trapping PARP complexes on DNA damage [25]. In summary, we suggest that several DDR factors function to mediate both HR and Alt-EJ, which may be important for cellular response to DNA damage not readily repaired by c-NHEJ, but that Alt-EJ has multiple mechanistic requirements that are distinct from HR.

Finally, we also examined the effect of the proteosome inhibitor Bortezomib on DSB repair, since this small molecule has been identified as an inhibitor of FANC signaling and HDR, and hence has been proposed as a DNA crosslink sensitizer for FANC proficient tumors [41, 63, 64]. Our findings support this concept, since we confirmed that Bortezomib inhibits FANC signaling. However, we also found that this small molecule substantially inhibits end resection, and furthermore causes a specific decrease in Alt-EJ and HR, relative to Distal-EJ. We suggest that the influence of Bortezomib on the DDR, and hence on DNA crosslink sensitivity, may not be limited to disrupting FANC signaling, but could include blocking Alt-EJ, HR, and/or end resection. In general, we suggest that blocking the initiation of not only HR, but also Alt-EJ, should be considered in developing therapeutics for DNA crosslink sensitization.

## Materials and Methods

### Cell lines, siRNA and plasmids

Establishment and culturing of U2OS reporter cell lines were described previously [42]. The WT mES cell line was acquired from ATCC (J1 strain), and the *Fanca^−/−^* cell line was generously provided by Drs. Maria Jasin and Koji Nakanishi [53, 69], and the *Ku70^−/−^* EJ2-GFP reporter cell line was described previously [12, 55]. The *Fanca* RT-PCR genotyping primers were 5’gtgtggtcggtggatgagat and 5’aacagctgaggctcctggta. The mES cells were cultured and used for integration of the EJ2-GFP and EJ5-GFP reporters at the *pim1* locus as described [12]. The *Fanca^−/−^Ku70^−/−^* EJ2-GFP cell line was generated using the expression plasmid pX330 [54] (generously deposited by Dr. Feng Zhang, Addgene 42230) for the CAS9 nuclease and gRNA with the following sequence introduced at the 5’ end of the gRNA: 5’gccatgggggtcgtcttcat, which targets a site in exon 5 of *Ku70*. This CAS9/Ku70-gRNA plasmid (0.5 μg) was co-transfected with a dsRED expression plasmid (0.1 μg, Clontech) into the *Fanca^−/−^* EJ2-GFP cell line using Lipofectamine 2000 (Invitrogen, 1.8 μl, total transfection volume 0.6 ml), and subsequently dsRED+ cells were sorted by fluorescence activated cell sorting, and seeded to isolate individual colonies. The genomic DNA from individual colonies was examined by PCR amplification (Platinum Hi-Fi Supermix, Invitrogen), using Kuex4UP 5’agatttggacaacccaggtc and Kuex5DN 5’gaggtcgctggctttggt, and *Bbs*1 digestion analysis to identify clones with mutations in the *Bbs*1 recognition site. Sequencing analysis of *Bbs*1 resistant PCR products identified a heterozygous clone with a frameshift mutation, which was re-transfected with dsRED and CAS9 plasmids and analyzed as above to identify a clone with loss of the *Bbs*1 site in the second allele that also caused a frameshift mutation, as determined by subcloning of the PCR product for sequencing analysis.

The library of pools of 4 siRNAs targeting 238 DDR factors is based on a commercially designed library (Dharmacon, G-006005). The sequence of each siRNA shown in Figs 1 and 3 are listed in S1 Table. The pCAGGS-BSKX empty vector and expression vectors for Ku70, I-SceI (pCBASce), and GFP (pCAGGS-NZE-GFP) were described previously [12]. The Fanca expression vector was generated by inserting the coding sequence from IMAGE clone 9087616 (Open Biosystems) into pCAGGS-BSKX. The 3xFlag-DNA2 vector was derived from pBABE-3xFlag-DNA2 (generously deposited by Dr. Sheila Stewart, Addgene 31955) [59] by introducing silent mutations for resistance to siDNA2–4 (5’TGATATcGAcACtCCtcTA), and subcloning into pMX-IRES-neo (Cell Biolabs). These pMX vectors were transfected with packaging plasmids (Addgene 8454 and 8449) [83] into 293T/17 cells (ATCC), and media from these transfected cells was filtered (0.45 μm) and used to treat the U2OS EJ2-GFP cell line, and integrants were subsequently selected using G418 (0.5 mg/ml).

### Transfections, DSB reporter assays, IRIF, and qRT-PCR analysis

Similar siRNA transfections were used for both DSB reporter assays and IRIF analysis: 0.5 × 10^5^ U2OS cells were plated in 0.5 ml antibiotic-free media on a 24 well plate with 5 pmol of each siRNA incubated with 1.8 μl RNAiMAX (Invitrogen), and cultured overnight (20 hrs). For the reporter assays, following the overnight RNAi treatment, cells were transfected with 0.5 μg of the I-SceI expression vector (pCBASce) using 1.8 μl Lipofectamine 2000 in 0.6 ml antibiotic-free media. The transfection media was removed after 3 hrs and replaced with antibiotic media. For mES cells, 0.3 × 10^5^ cells were plated 20 hours prior to transfection with 0.4 μg I-SceI and 0.2 μg of a second vector (expression vector for Fanca, Ku70, or EV). Transient GFP expression transfections used 0.4 μg of pCAGGS-NZE-GFP in place of pCBASce. For drug treatment, Olaparib (AZD2281, Selleck Chemicals, S1060), Bortezomib (Santa Cruz Biotech, sc-217785), or vehicle (DMSO) was added immediately after removing the transfection complexes (4 hrs for mES, 3 hrs for U2OS) for a continued treatment for the rest of the experiment. Three days after the plasmid transfections, GFP+ frequencies were determined by flow cytometery using a CyAn ADP Analyzer (Beckman Coulter, Inc.), as described [42]. The GFP+ frequencies shown relative to a control sample were calculated by dividing the GFP+ frequency for each transfection by the mean value for the control samples treated in parallel (siCTRL, EV, and/or DMSO treated). Each repair value is the mean of at least three independent transfections, error bars reflect the standard deviation, and statistics were performed with the unpaired *t*-test. Error bars denote the standard deviation from the mean.

For IRIF analysis, following overnight siRNA treatment (20 hrs), cells were plated onto chamber slides and cultured for a second day before IR treatment. For experiments without siRNA, cells were plated directly on chamber slides, and the next day were pre-treated with 100 nM Bortezomib or vehicle (DMSO) for 4 hrs prior to IR. Slides were treated with 10 Gy of IR (Gammacell 3000), allowed to recover for 4 hr (with Bortezomib or vehicle when appropriate), fixed with 4% paraformaldehyde and treated with 0.1 M glycine and 0.5% triton-X 100 prior to probing with antibodies against γH2AX (Abcam, ab18311) and FANCD2 (Abcam, ab2187), followed by secondary antibodies (Invitrogen/Life Technologies, A-11036 and A11029), and with DAPI using Vectashield Mounting Medium (Vector Laboratories). Images were acquired using a BX-50 (Olympus) microscope with Image-Pro software. At least 100 cells that showed γH2AX foci were scored for >20 FANCD2 IRIF from at least three independent treatments per condition. Statistics were performed as for the reporter assays.

For qRT-PCR analysis to examine RNAi depletion of target mRNAs, total RNA was isolated 2 days after siRNA transfection (Qiagen RNAeasy Plus) and reverse transcribed with MMLV-RT (Promega). The RT reactions were amplified with primers for the target mRNA (primers in S1 Table) and actin (5’actgggacgacatggagaag and 5’aggaaggaaggctggaagag) using SYBR Select Master Mix (Life Technologies) and quantified on a ViiA 7 Real Time PCR System (Life Technologies). Fold depletion for each siRNA treatment was determined as 2^ΔΔCt^, for which the cycle threshold (Ct) value for the target mRNA was subtracted by Ct value for actin (mean of duplicate amplifications from the same RT reaction) to calculate the ΔCt value, which was then subtracted from the corresponding ΔCt from siCTRL treated cells to calculate ΔΔCt.

### Immunoblotting analysis

Cells were lysed with NETN (20 mM Tris pH 8, 100 mM NaCl, 1 mM EDTA, 0.5% IGEPAL, 1.25 mM DTT and Roche Protease Inhibitor) or RIPA (Sigma R0278 with 1.25 mM DTT and Roche Protease Inhibitor Cocktail, Roche PhosStop for U2OS, for PAR analysis), and using several freeze/thaw cycles or sonication (QSonica Q800RS ultrasonic horn). Blots of these extracts were probed with antibodies against CtIP (Santa Cruz Biotech, sc-5970), FANCA (Bethyl Laboratories, Inc., A301–980A to detect human FANCA, and Santa Cruz Biotech, sc-23612 to detect expression of mouse Fanca), FANCD2 (Abcam, ab2187), Ku70 (Santa Cruz Biotech, sc-1487), PAR (Trevigen, 4335-MC-100), Flag (Sigma, A8592), actin (Sigma, A2066), and HRP-conjugated secondary antibodies (Santa Cruz Biotech, sc-2004 and sc-2005). ECL reagent (Amersham Biosciences) was used to visualize HRP signals.

### Clongenic survival assays

For cisplatin sensitivity, mES cells were plated one day prior to continual exposure to 0.12 μM or 0.25 μM cisplatin (Pfizer NOC-0069-0081-01) for one week to form colonies. For IR sensitivity, mES cells were treated in suspension with 1 Gy or 3 Gy IR (Gammacell 3000) and plated for one week to form colonies. Colonies were fixed (10% Acetic Acid, 10% Methanol), stained with 1% crystal violet, and counted under the miscroscope (10X). To calculate fraction clonogenic survival, the number of colonies per well were normalized to the number of cells plated, and this colony forming value for each treated well was divided by the mean value of parallel untreated plates. Each clonogenic survival value represents the mean of at least six independent treatments, and error bars denote the standard deviation.

### End resection and cell cycle analysis

Cells were treated with siRNAs as for the reporter assays, but scaled up to one well of a 6 well plate, and cultured for two days prior to either end resection and cell cycle analysis. For drug treatments, 100 nM bortezomib and 5 μM Olaparib were added prior to performing the assays (4 hr and 20 hr, respectively). For the end resection assay, cells were treated with 1μM camptothecin for 1 hr prior to harvesting for RPA and DAPI staining, as described [61]. Briefly, cell pellets were detergent extracted in 100 μl of 0.2% Triton X-100 in PBS on ice for 7 minutes, and washed with 1 ml of BSA-PBS (0.1% BSA in PBS). Next, cells were fixed with 100 μl BD Cytofix/Cytoperm buffer (BD Biosciences) for 15 min, washed with BSA-PBS, and incubated in 50 μl of BD Perm/Wash buffer (BD Biosciences) with 1:200 RPA antibody (RPA2 9H8 Abcam ab2175) for 1 hour. Cells were washed with BSA-PBS and re-suspended in 50 μl BD Perm/Wash buffer with 1:200 secondary antibody (goat anti mouse Alexa Fluor 488, Life Technologies A11029) for 30 min. Finally, cells were washed with BSA-PBS and re-suspended in 0.3 ml PBS with 0.02% sodium azide, 250 μg/ml RNase A (Sigma R4642) and 2 μg/ml DAPI (Sigma D8417) for 30 min at 37°C. For cell cycle analysis, cells were incubated with 10 mM bromodeoxyuridine (BrdU, Sigma B5002) for 30 min prior to harvesting, fixed in 70% ethanol, stained with FITC-conjugated anti-BrdU antibody (BD Biosciences, 51–33284X), and incubated with propidium iodide (PI, Sigma P4170) and RNase A (Sigma R4642). Stained cells for both assays were analyzed on a CyAn ADP Flow Cytometer.

## Supporting Information

S1 Table Shown are siRNA and qRT-PCR primer sequences. The siRNA pools are derived from a mixture of the four siRNAs listed. Also shown is the siRNA number as referenced in the text, which can be distinct from the catalog number.
(DOC)

S2 Table Shown are the results of the screen of 238 siRNA pools with the EJ2-GFP (Alt-EJ) and EJ5-GFP (Distal-EJ) reporter assays. Shown are the links to the NCBI Gene webpage and gene name for each siRNA target of the screen. We determined the fold change caused by each siRNA pool on both reporters (N = 2) relative to parallel siCTRL treatments, which we used to calculate the ratio of the fold change on Alt-EJ *versus* Distal-EJ. We then performed additional repeats of several siRNA pools that appeared to cause the greatest effects on the Alt-EJ/Distal-EJ ratio, and then ranked the siRNA pools according to Alt-EJ/Distal-EJ ratio to complete the screen. The *N* column refers to the number of times each siRNA pool was examined in the screen. The fold change column refers to the average repair value for each siRNA pool relative to parallel siCTRL treatments. The Alt-EJ/Distal-EJ column indicates the Alt-EJ fold change value divided by the Distal-EJ fold change value.
(DOC)

S1 Fig Ratio of the fold change on Alt-EJ *versus* Distal-EJ, relative to siCTRL is shown. (**A**) for each siRNA in the screen (S2 Table), and (**B**) for each siRNA shown in Fig. 1C.Since each fold change is calculated relative to siCTRL, the ratio for siCTRL = 1.
(PDF)

S2 Fig End resection analysis. (**A**) Shown are representative flow cytometry plots from cells treated with siCTRL without camptothecin (siCTRL UN), siCTRL with camptothecin (siCTRL 1μM CPT) and siCtIP with camptothecin (CtIP-1 1μM CPT). U2OS cells were treated and analyzed for RPA staining as described in Fig. 6E. (**B**) Analysis of the effects of siRNA treatment on end resection. Shown is the percentage of cells with RPA staining after siRNA treatment using siRNA pools targeting the genes shown (i.e. the genes from Fig. 2C, except DNA2 and FANCA, which are shown in Fig. 6F).
(PDF)

S3 Fig Shown are representative images of U2OS cells treated with Bortezomib (100 nM, 4 hr before IR treatment) or vehicle (DMSO) as well as U2OS cells treated with an individual FANCA siRNA (siFANCA-3) or non-targeting siRNA (siCTRL). Cells were treated with 10 Gy IR and allowed to recover (4 hr) prior to fixation and immunostaining for FANCD2 and the DSB marker γH2AX. *indicates representative cells with >20 FANCD2 foci.
(PDF)
