# Supplementary material for: DNA Damage Response Factors from Diverse Pathways, Including DNA Crosslink Repair, Mediate Alternative End Joining
Source: PLoS Genet. 2015 Jan 28;11(1):e1004943. doi: 10.1371/journal.pgen.1004943 (PMC4309583; doi:10.1371/journal.pgen.1004943)
Supplement: S1 Table — The siRNA pools are derived from a mixture of the four siRNAs listed. Also shown is the siRNA number as referenced in the text, which can be distinct from the catalog number. (DOC) [file pgen.1004943.s001.doc]

**Supplementary Table S1**

| **siRNA** | **Catalog #** | **Target Sequence** | **RT-PCR forward primer** | **RT-PCR reverse primer** |
| --- | --- | --- | --- | --- |
| FANCA | D-019283-19 | CGACAUGCAUGCUGUGGGA | ATAGGCTCTGCTTTGCAGGA | GCAGGATGCTTCCATCTGTT |
|  | D-019283-20 | CGCUUUGGCUGCUGGAGUA |  |  |
|  | D-019283-21 | GGACAGAUCUGCACGGCUC (FANCA #3) |  |  |
|  | D-019283-22 | GUUAGAGUUUGCUCAGUAU (FANCA#4) |  |  |
| FAAP24 | D-016958-01 | CCAAAGAGCCCAGUAAGAA (FAAP24 #1) | GGATGGCTTGACACCAGACT | GGAGGAGAAGGGGAGCTTTA |
|  | D-016958-02 | UAAAGGAAUUGUAGUCGUU |  |  |
|  | D-016958-03 | CAGGAAAUGGCUACAGAAA (FAAP24 #3) |  |  |
|  | D-016958-04 | CGGGUUAGAAAUUCCAAUA |  |  |
| NTHL1 | D-009345-01 | GGAGCAAGGUGAAAUACAU | AAACCAAAGACCAGGTGACG | CCCACCAAGAGTCCATTGAT |
|  | D-009345-02 | GGACCAAGAAGGCAACCAA (NTHL1 #2) |  |  |
|  | D-009345-03 | CUGAUGCUCUCCAGCCAAA |  |  |
|  | D-009345-04 | GGAUGCACCUGUGGACCAU (NTHL1 #4) |  |  |
| UNG | D-011795-01 | CUACAGACAUAGAGGAUUU | CCCATCAAGCCAACTCTCAT | GAAGCTTTTCCCCCTTATGC |
|  | D-011795-02 | GAACUCGAAUGGCCUUGUU (UNG #2) |  |  |
|  | D-011795-03 | GCAGUGCCAUUGAUAGGAA (UNG#3) |  |  |
|  | D-011795-04 | CAAGCCAACUCUCAUAAGG (UNG #4) |  |  |
| SOD1 | D-008364-05 | UCGUUUGGCUUGUGGUGUA | AGGGCATCATCAATTTCGAG | TTACACCACAAGCCAAACGA |
|  | D-008364-06 | ACAAAGAUGGUGUGGCCGA (SOD1 #2) |  |  |
|  | D-008364-07 | GUGCAGGGCAUCAUCAAUU (SOD1 #3) |  |  |
|  | D-008364-08 | UUAAUCCUCUAUCCAGAAA (SOD1 #4) |  |  |
| RUVBL2 | D-012299-01 | UAACAAGGAUUGAGCGAAU | CCCCTACAGCGAGAAAGACA | GCAGCTTCCCAACCATTTTA |
|  | D-012299-02 | CGCAGUACAUGAAGGAGUA (RUVBL2 #2) |  |  |
|  | D-012299-03 | UCAACGAACUCAAAGGCGA |  |  |
|  | D-012299-04 | ACGCAAGGGUACAGAAGUG (RUVBL2 #4) |  |  |
| TIP60 | D-006301-01 | GGACAGCUCUGAUGGAAUA (TIP60 #1) | CATCCTCCAGGCAATGAGAT | GTCTGGGACCAGTAGCTTCG |
|  | D-006301-05 | CACAGGAACUCACCACAUU |  |  |
|  | D-006301-06 | GAACAAGAGUUAUUCCCAG (TIP60 #3) |  |  |
|  | D-006301-07 | CCACAGAUCACCAUCAAUG (TIP60 #4) |  |  |
| RAD23B | D-011759-01 | GCAGAUAGGUCGAGAGAAU (RAD23B #1) | CAGCAGAATCCTTCCTTGCT | TGCACTGGTGTGAAGTGTGA |
|  | D-011759-02 | GUACAUCGGGUGAUUCUUC |  |  |
|  | D-011759-03 | GAACGAGAGCAAGUAAUUG (RAD23B #3) |  |  |
|  | D-011759-04 | GGGUCAGUCUUACGAGAAU |  |  |
| GEN1 | D-018757-01 | GUAAAGACCUGCAAUGUUA (GEN1 #1) | TGGGATTAGTCCTGAAGAGCA | ATTTTCCGAGTGGTTTGCAT |
|  | D-018757-02 | UCUAAGACCUUUGGCUAUA (GEN1 #2) |  |  |
|  | D-018757-03 | GUAAUGAGCUUUCAGUCAC |  |  |
|  | D-018757-04 | GAAUCUAGUCAACCCAAUA (GEN1 #4) |  |  |
| DNA2 | D-026431-01 | GCUAAACCGUGAAGCAAGA | ACATGGTGCCATACCTGTCA | CTTTTTCATCGCTTGCCTCT |
|  | D-026431-02 | CUACGUCACUUUAAAGAUG (DNA2 #2) |  |  |
|  | D-026431-03 | ACAGUUGCCUGCAUUCUAA (DNA2 #3) |  |  |
|  | D-026431-04 | UGAUAUAGAUACCCCAUUA (DNA2 #4) |  |  |
| MSH6 | D-019287-01 | GUAGAAAGAUGGCACAUAU | GGGATCATGGAAGAAGTTGC | TCGTTTACAGCCCTTCTTGG |
|  | D-019287-02 | CGAAGUAGCCGCCAAAUAA |  |  |
|  | D-019287-03 | GAACAGAGCCUCCUGGAAU (MSH6 #3) |  |  |
|  | D-019287-04 | GCUCUGAUGUGGAAUUUAA (MSH6 #4) |  |  |
| PRP19 | D-004668-01 | CAGAAGAGCUCAGCAAAUA (PRP19 #1) | GGAAGACTGTGCCTGAGGAG | TCGGAGGAGCTCAGGAGATA |
|  | D-004668-02 | GAUAACAACUUUGAGGUAA |  |  |
|  | D-004668-04 | GAUCUGCGCAAGCUUAAGA |  |  |
|  | D-004668-17 | CUAACGAAGUGCCGGAGCA (PRP19 #4) |  |  |
| POLA1 | D-020856-01 | GCAGUAACAUCGAUUGUAA (POLA1 #1) | GCTGCCTTGGTGACATACAA | CCGGCTTTGATCAGAAAGAA |
|  | D-020856-02 | GACAUUAGACGUUUCAUUA (POLA1 #2) |  |  |
|  | D-020856-03 | UAACAUCGCUGGGAACAUU (POLA1 #3) |  |  |
|  | D-020856-04 | GCUCAAAGGAUUAGAUAUA |  |  |
| CtIP | D-011376-01 | GAGCAGACCUUUCUCAGUA |  |  |
|  | D-011376-02 | GAAGUGAACAAGAUCAUUA |  |  |
|  | D-011376-03 | CAACCAAGAUGUAUCCUUU |  |  |
|  | D-011376-04 | GAAUAGGACUGAGUACGGU |  |  |
|  | AM16104 | GCUAAAACAGGAACGAAUC (CtIP-1) |  |  |
| FANCD2 | D-016376-01 | GGUCAGAGCUGUAUUAUUC | TGGCTTGACAGAGTTGTGGA | GTGATGGCAAAACACAATGC |
|  | D-016376-02 | GAUAAGUUGUCGUCUAUUA |  |  |
|  | D-016376-04 | GAUCAACUCUCCUAAAGAU |  |  |
|  | D-016376-18 | GAACAAAGGAAGCCGGAAU |  |  |
| FANCC | D-011033-01 | GGUAUGCACCUAUAGAUUA | ATCAGGCACCTTCTCCTCAA | GACCTGGCTCTGCATTTTGT |
|  | D-011033-02 | GACCAGACCUUGUACAGAU |  |  |
|  | D-011033-03 | GGAAUCGUCUUGGCAUUGA |  |  |
|  | D-011033-04 | GAGAGAAUCAUCUUAAUGG |  |  |
| FANCE | D-013991-01 | CAACUGCCCUGACCUCCUU | GGGAGCTTCTCCACTGTCTG | GATAGCTTTGGGCAACTCCA |
|  | D-013991-02 | GGAGAGACCCGAACAUAAG |  |  |
|  | D-013991-03 | CCAAGUAUCAGGCUAACAU |  |  |
|  | D-013991-04 | CGAAUCUGGAUGAUGCUAA |  |  |
| FANCF | D-014206-01 | GGGAGGAGUUGCACAAUAG | GCTAGTCCACTGGCTTCTGG | CGCTGAGACCCAAAACTTGT |
|  | D-014206-02 | GGUCAACGUUUGCACUAUG |  |  |
|  | D-014206-03 | UAACUGCCCUGGAGACCUG |  |  |
|  | D-014206-04 | CUUAUUAGCUCUUCGUAGU |  |  |
| FANCG | D-011899-01 | GGAAUUAGAUGCUCCAUUG | TGTCCTCCTGACAGCATTTG | ATGAAGGGGTGAGGCTAGGT |
|  | D-011899-02 | GGACAUCUCUGCCAAAGUC |  |  |
|  | D-011899-03 | GCAAGCAGGUGCCUACAGA |  |  |
|  | D-011899-04 | GCACUAAGCAGCCUUCAUG |  |  |
| FANCI | D-022320-01 | AGACCUAGAUGAUAUAUUG | GGAGGAGGAAGAGGCATTCT | AAGGCTTTCTTGGTGGCTTT |
|  | D-022320-02 | GUAAGAGCCUGAACUAUAC |  |  |
|  | D-022320-03 | GAUGGGAGUUUGUGAGGUU |  |  |
|  | D-022320-04 | ACUCUCAGCUGGCUAAUUC |  |  |
| FANCL | D-021486-01 | GAAGUUGCCUUAAAGAAUA | GTGTTTCTCCGGACTTCGAG | GCAGGTATCCGCATACACAA |
|  | D-021486-02 | GCGGAUACCUGCUUCAGUA |  |  |
|  | D-021486-03 | GAACAUACAUUUGUGGGAU |  |  |
|  | D-021486-04 | GAUAGGAACUCUUGGUUGG |  |  |
| FANCM | D-021955-01 | GUACUGCACUUGAGAAUUU | GTCAAAAAGCGCAGATTTCC | TCAGGAATCTGCGAGAAAATG |
|  | D-021955-02 | CAAACCAUGUUCACAAUUA |  |  |
|  | D-021955-03 | CAACAGUGGUGAAUAGUAA |  |  |
|  | D-021955-04 | GAACAAGAUUCCUCAUUAC |  |  |
