# Supplementary material for: DNA Damage Response Factors from Diverse Pathways, Including DNA Crosslink Repair, Mediate Alternative End Joining
Source: PLoS Genet. 2015 Jan 28;11(1):e1004943. doi: 10.1371/journal.pgen.1004943 (PMC4309583; doi:10.1371/journal.pgen.1004943)
Supplement: S2 Table — Shown are the links to the NCBI Gene webpage and gene name for each siRNA target of the screen. We determined the fold change caused by each siRNA pool on both reporters (N = 2) relative to parallel siCTRL treatments, which we used to calculate the ratio of the fold change on Alt-EJ versus Distal-EJ. We then performed additional repeats of several siRNA pools that appeared to cause the greatest effects on the Alt-EJ/Distal-EJ ratio, and then ranked the siRNA pools according to Alt-EJ/Distal-EJ ratio to complete the screen. The N column refers to the number of times each siRNA pool was examined in the screen. The fold change column refers to the average repair value for each siRNA pool relative to parallel siCTRL treatments. The Alt-EJ/Distal-EJ column indicates the Alt-EJ fold change value divided by the Distal-EJ fold change value. (DOC) [file pgen.1004943.s002.doc]

**Supplementary** Table S2

| **Link** | **siRNA Treatment** | **Fold Change (EJ2/Alt-EJ)** | ***N***  **(EJ2/Alt-EJ)** | **Fold Change (EJ5/Distal-EJ)** | ***N***  **(EJ5/Distal-EJ)** | **Alt-EJ/Distal-EJ** |
| --- | --- | --- | --- | --- | --- | --- |
| <http://www.ncbi.nlm.nih.gov/gene/1763> | DNA2 | 0.19 | 10 | 0.44 | 6 | 0.44 |
| <http://www.ncbi.nlm.nih.gov/gene/6647> | SOD1 | 0.32 | 6 | 0.70 | 6 | 0.46 |
| <http://www.ncbi.nlm.nih.gov/gene/4913> | NTHL1 | 0.30 | 6 | 0.60 | 6 | 0.50 |
| <http://www.ncbi.nlm.nih.gov/gene/91442> | FAAP24 | 0.33 | 8 | 0.66 | 8 | 0.50 |
| <http://www.ncbi.nlm.nih.gov/gene/5422> | POLA1 | 0.40 | 6 | 0.80 | 6 | 0.50 |
| <http://www.ncbi.nlm.nih.gov/gene/7374> | UNG | 0.46 | 10 | 0.91 | 6 | 0.51 |
| <http://www.ncbi.nlm.nih.gov/gene/10524> | TIP60 | 0.24 | 10 | 0.45 | 6 | 0.54 |
| <http://www.ncbi.nlm.nih.gov/gene/348654> | GEN1 | 0.39 | 10 | 0.72 | 6 | 0.54 |
| <http://www.ncbi.nlm.nih.gov/gene/27339> | PRP19 | 0.49 | 6 | 0.84 | 6 | 0.58 |
| <http://www.ncbi.nlm.nih.gov/gene/2475> | MTOR | 0.47 | 6 | 0.80 | 6 | 0.58 |
| <http://www.ncbi.nlm.nih.gov/gene/2968> | GTF2H4 | 0.52 | 6 | 0.88 | 6 | 0.59 |
| <http://www.ncbi.nlm.nih.gov/gene/51750> | RTEL1 | 0.53 | 6 | 0.89 | 6 | 0.59 |
| <http://www.ncbi.nlm.nih.gov/gene/2956> | MSH6 | 0.49 | 6 | 0.83 | 6 | 0.59 |
| <http://www.ncbi.nlm.nih.gov/gene/5887> | RAD23B | 0.51 | 6 | 0.86 | 6 | 0.60 |
| <http://www.ncbi.nlm.nih.gov/gene/10856> | RUVBL2 | 0.45 | 10 | 0.73 | 6 | 0.62 |
| <http://www.ncbi.nlm.nih.gov/gene/328> | APEX1 | 0.41 | 6 | 0.64 | 6 | 0.63 |
| <http://www.ncbi.nlm.nih.gov/gene/5427> | POLE2 | 0.64 | 2 | 0.98 | 2 | 0.66 |
| <http://www.ncbi.nlm.nih.gov/gene/2177> | FANCD2 | 0.54 | 6 | 0.82 | 6 | 0.66 |
| <http://www.ncbi.nlm.nih.gov/gene/79035> | NABP2 | 0.49 | 2 | 0.74 | 2 | 0.66 |
| <http://www.ncbi.nlm.nih.gov/gene/10519> | CIB1 | 0.42 | 2 | 0.62 | 2 | 0.67 |
| <http://www.ncbi.nlm.nih.gov/gene/353497> | POLN | 0.60 | 2 | 0.89 | 2 | 0.67 |
| <http://www.ncbi.nlm.nih.gov/gene/8045> | HRAS1 | 0.40 | 2 | 0.59 | 2 | 0.68 |
| <http://www.ncbi.nlm.nih.gov/gene/2071> | XPB | 0.63 | 10 | 0.93 | 6 | 0.68 |
| <http://www.ncbi.nlm.nih.gov/gene/5892> | RAD51D | 0.44 | 6 | 0.64 | 6 | 0.68 |
| <http://www.ncbi.nlm.nih.gov/gene/7319> | UBE2A | 0.53 | 2 | 0.77 | 2 | 0.69 |
| <http://www.ncbi.nlm.nih.gov/gene/8438> | RAD54L | 0.52 | 2 | 0.76 | 2 | 0.69 |
| <http://www.ncbi.nlm.nih.gov/gene/4287> | ATXN3 | 0.60 | 6 | 0.86 | 6 | 0.69 |
| <http://www.ncbi.nlm.nih.gov/gene/5890> | RAD51B | 0.55 | 2 | 0.79 | 2 | 0.70 |
| <http://www.ncbi.nlm.nih.gov/gene/23064> | SETX | 0.64 | 2 | 0.91 | 2 | 0.70 |
| <http://www.ncbi.nlm.nih.gov/gene/4221> | MEN1 | 0.45 | 6 | 0.64 | 6 | 0.70 |
| <http://www.ncbi.nlm.nih.gov/gene/9937> | PSO2 | 0.64 | 2 | 0.91 | 2 | 0.70 |
| <http://www.ncbi.nlm.nih.gov/gene/51455> | REV1 | 0.66 | 2 | 0.94 | 2 | 0.70 |
| <http://www.ncbi.nlm.nih.gov/gene/11044> | PAPD7 | 0.51 | 2 | 0.72 | 2 | 0.70 |
| <http://www.ncbi.nlm.nih.gov/gene/2175> | FANCA | 0.57 | 6 | 0.81 | 6 | 0.71 |
| <http://www.ncbi.nlm.nih.gov/gene/6119> | RPA3 | 0.57 | 2 | 0.81 | 2 | 0.71 |
| <http://www.ncbi.nlm.nih.gov/gene/27301> | APE2 | 0.46 | 6 | 0.64 | 6 | 0.72 |
| <http://www.ncbi.nlm.nih.gov/gene/56852> | RAD18 | 0.54 | 2 | 0.75 | 2 | 0.72 |
| <http://www.ncbi.nlm.nih.gov/gene/64858> | APOLLO | 0.56 | 2 | 0.77 | 2 | 0.72 |
| <http://www.ncbi.nlm.nih.gov/gene/4438> | MSH4 | 0.55 | 2 | 0.77 | 2 | 0.72 |
| <http://www.ncbi.nlm.nih.gov/gene/5932> | RBBP8 | 0.59 | 10 | 0.82 | 6 | 0.72 |
| <http://www.ncbi.nlm.nih.gov/gene/10039> | PARP3 | 0.46 | 2 | 0.64 | 2 | 0.73 |
| <http://www.ncbi.nlm.nih.gov/gene/4968> | OGG1 | 0.61 | 2 | 0.84 | 2 | 0.73 |
| <http://www.ncbi.nlm.nih.gov/gene/10155> | KAP1 | 0.56 | 2 | 0.77 | 2 | 0.73 |
| <http://www.ncbi.nlm.nih.gov/gene/57697> | FANCM | 0.45 | 2 | 0.62 | 2 | 0.73 |
| <http://www.ncbi.nlm.nih.gov/gene/252969> | NEIL2 | 0.56 | 6 | 0.77 | 6 | 0.73 |
| <http://www.ncbi.nlm.nih.gov/gene/1032> | CDKN2D | 0.49 | 2 | 0.67 | 2 | 0.73 |
| <http://www.ncbi.nlm.nih.gov/gene/54840> | APTX | 0.64 | 2 | 0.86 | 2 | 0.74 |
| <http://www.ncbi.nlm.nih.gov/gene/4521> | MTH1 | 0.48 | 2 | 0.64 | 2 | 0.74 |
| <http://www.ncbi.nlm.nih.gov/gene/27434> | POLM | 0.49 | 2 | 0.65 | 2 | 0.74 |
| <http://www.ncbi.nlm.nih.gov/gene/51426> | POLK | 0.53 | 2 | 0.71 | 2 | 0.75 |
| <http://www.ncbi.nlm.nih.gov/gene/5429> | POLH | 0.57 | 6 | 0.77 | 6 | 0.75 |
| <http://www.ncbi.nlm.nih.gov/gene/3980> | LIG3 | 0.66 | 6 | 0.88 | 6 | 0.75 |
| <http://www.ncbi.nlm.nih.gov/gene/4439> | MSH5 | 0.53 | 2 | 0.71 | 2 | 0.75 |
| <http://www.ncbi.nlm.nih.gov/gene/7486> | WRN | 0.41 | 6 | 0.55 | 6 | 0.75 |
| <http://www.ncbi.nlm.nih.gov/gene/79728> | PALB2 | 0.39 | 2 | 0.52 | 2 | 0.75 |
| <http://www.ncbi.nlm.nih.gov/gene/4904> | YBX1 | 0.58 | 6 | 0.76 | 6 | 0.76 |
| <http://www.ncbi.nlm.nih.gov/gene/7334> | UBC13 | 0.64 | 2 | 0.84 | 2 | 0.76 |
| <http://www.ncbi.nlm.nih.gov/gene/11073> | TOPBP1 | 0.62 | 2 | 0.81 | 2 | 0.76 |
| <http://www.ncbi.nlm.nih.gov/gene/2178> | FANCE | 0.80 | 2 | 1.05 | 2 | 0.76 |
| <http://www.ncbi.nlm.nih.gov/gene/91419> | XRCC6BP1 | 0.84 | 2 | 1.10 | 2 | 0.76 |
| <http://www.ncbi.nlm.nih.gov/gene/25842> | ASF1A | 0.48 | 2 | 0.63 | 2 | 0.77 |
| <http://www.ncbi.nlm.nih.gov/gene/9025> | RNF8 | 0.57 | 2 | 0.74 | 2 | 0.77 |
| <http://www.ncbi.nlm.nih.gov/gene/10459> | MAD2L2 | 0.50 | 2 | 0.64 | 2 | 0.78 |
| <http://www.ncbi.nlm.nih.gov/gene/6419> | METNASE | 0.62 | 2 | 0.80 | 2 | 0.78 |
| <http://www.ncbi.nlm.nih.gov/gene/2547> | XRCC6 | 0.70 | 2 | 0.90 | 2 | 0.78 |
| <http://www.ncbi.nlm.nih.gov/gene/1453> | CSNK1D | 0.51 | 6 | 0.65 | 6 | 0.78 |
| <http://www.ncbi.nlm.nih.gov/gene/1454> | CSNK1E | 0.66 | 2 | 0.84 | 2 | 0.78 |
| <http://www.ncbi.nlm.nih.gov/gene/7516> | XRCC2 | 0.66 | 2 | 0.84 | 2 | 0.79 |
| <http://www.ncbi.nlm.nih.gov/gene/3014> | H2AX | 0.52 | 2 | 0.66 | 2 | 0.79 |
| <http://www.ncbi.nlm.nih.gov/gene/7336> | MMS2 | 0.62 | 2 | 0.78 | 2 | 0.79 |
| <http://www.ncbi.nlm.nih.gov/gene/7979> | SHFM1 | 0.71 | 2 | 0.90 | 2 | 0.79 |
| <http://www.ncbi.nlm.nih.gov/gene/5383> | PMS2P5 | 0.70 | 2 | 0.89 | 2 | 0.79 |
| <http://www.ncbi.nlm.nih.gov/gene/9577> | BRCC4 | 0.71 | 2 | 0.89 | 2 | 0.79 |
| <http://www.ncbi.nlm.nih.gov/gene/5423> | POLB | 0.60 | 2 | 0.75 | 2 | 0.80 |
| <http://www.ncbi.nlm.nih.gov/gene/9400> | RECQ5 | 0.55 | 6 | 0.69 | 6 | 0.80 |
| <http://www.ncbi.nlm.nih.gov/gene/55775> | TDP1 | 0.70 | 2 | 0.88 | 2 | 0.80 |
| <http://www.ncbi.nlm.nih.gov/gene/3148> | HMGB2 | 0.62 | 2 | 0.77 | 2 | 0.80 |
| <http://www.ncbi.nlm.nih.gov/gene/51567> | TDP2 | 0.60 | 2 | 0.75 | 2 | 0.80 |
| <http://www.ncbi.nlm.nih.gov/gene/2176> | FANCC | 0.53 | 2 | 0.65 | 2 | 0.81 |
| <http://www.ncbi.nlm.nih.gov/gene/2965> | GTF2H1 | 0.78 | 2 | 0.96 | 2 | 0.81 |
| <http://www.ncbi.nlm.nih.gov/gene/5980> | REV3L | 0.68 | 2 | 0.84 | 2 | 0.81 |
| <http://www.ncbi.nlm.nih.gov/gene/50484> | RRM2B | 0.69 | 2 | 0.85 | 2 | 0.81 |
| <http://www.ncbi.nlm.nih.gov/gene/6118> | RPA2 | 0.62 | 2 | 0.77 | 2 | 0.81 |
| <http://www.ncbi.nlm.nih.gov/gene/4436> | MSH2 | 0.70 | 2 | 0.85 | 2 | 0.82 |
| <http://www.ncbi.nlm.nih.gov/gene/10038> | PARP2 | 0.74 | 2 | 0.90 | 2 | 0.82 |
| <http://www.ncbi.nlm.nih.gov/gene/55247> | NEIL3 | 0.74 | 2 | 0.90 | 2 | 0.82 |
| <http://www.ncbi.nlm.nih.gov/gene/9401> | RECQ4 | 0.58 | 2 | 0.71 | 2 | 0.82 |
| <http://www.ncbi.nlm.nih.gov/gene/902> | CCNH | 0.78 | 2 | 0.95 | 2 | 0.82 |
| <http://www.ncbi.nlm.nih.gov/gene/2966> | GTF2H2 | 0.74 | 2 | 0.90 | 2 | 0.82 |
| <http://www.ncbi.nlm.nih.gov/gene/10474> | TADA3 | 0.53 | 2 | 0.64 | 2 | 0.83 |
| <http://www.ncbi.nlm.nih.gov/gene/7517> | XRCC3 | 0.58 | 6 | 0.70 | 6 | 0.83 |
| <http://www.ncbi.nlm.nih.gov/gene/4292> | MLH1 | 0.79 | 2 | 0.96 | 2 | 0.83 |
| <http://www.ncbi.nlm.nih.gov/gene/5591> | DNA PKcs | 0.64 | 2 | 0.76 | 2 | 0.83 |
| <http://www.ncbi.nlm.nih.gov/gene/10111> | RAD50 | 0.88 | 2 | 1.04 | 2 | 0.84 |
| <http://www.ncbi.nlm.nih.gov/gene/5885> | SCC1 | 0.63 | 2 | 0.74 | 2 | 0.84 |
| <http://www.ncbi.nlm.nih.gov/gene/200558> | APLF | 0.70 | 2 | 0.83 | 2 | 0.84 |
| <http://www.ncbi.nlm.nih.gov/gene/5428> | POLG | 0.73 | 2 | 0.87 | 2 | 0.85 |
| <http://www.ncbi.nlm.nih.gov/gene/64421> | Artemis | 0.62 | 2 | 0.73 | 2 | 0.85 |
| <http://www.ncbi.nlm.nih.gov/gene/10309> | UNG2 | 0.62 | 2 | 0.73 | 2 | 0.85 |
| <http://www.ncbi.nlm.nih.gov/gene/5886> | RAD23A | 0.79 | 6 | 0.93 | 6 | 0.85 |
| <http://www.ncbi.nlm.nih.gov/gene/2648> | KAT2A | 0.74 | 2 | 0.87 | 2 | 0.86 |
| <http://www.ncbi.nlm.nih.gov/gene/55170> | PRMT6 | 0.63 | 6 | 0.73 | 6 | 0.86 |
| <http://www.ncbi.nlm.nih.gov/gene/5395> | PMS2 | 0.84 | 2 | 0.98 | 2 | 0.86 |
| <http://www.ncbi.nlm.nih.gov/gene/7398> | UBP | 0.58 | 2 | 0.67 | 2 | 0.86 |
| <http://www.ncbi.nlm.nih.gov/gene/2189> | FANCG | 0.68 | 2 | 0.79 | 2 | 0.86 |
| <http://www.ncbi.nlm.nih.gov/gene/136647> | MPLKIP | 0.60 | 2 | 0.70 | 2 | 0.86 |
| <http://www.ncbi.nlm.nih.gov/gene/9126> | SMC3 | 0.75 | 10 | 0.87 | 6 | 0.86 |
| <http://www.ncbi.nlm.nih.gov/gene/9787> | DLGAP5 | 0.68 | 2 | 0.79 | 2 | 0.87 |
| <http://www.ncbi.nlm.nih.gov/gene/4437> | MSH3 | 0.47 | 2 | 0.54 | 2 | 0.87 |
| <http://www.ncbi.nlm.nih.gov/gene/2072> | XPF | 0.75 | 2 | 0.86 | 2 | 0.87 |
| <http://www.ncbi.nlm.nih.gov/gene/2188> | FANCF | 0.68 | 2 | 0.78 | 2 | 0.87 |
| <http://www.ncbi.nlm.nih.gov/gene/56949> | XAB2 | 0.55 | 6 | 0.64 | 6 | 0.87 |
| <http://www.ncbi.nlm.nih.gov/gene/404672> | GTF2H5 | 0.62 | 10 | 0.72 | 6 | 0.87 |
| <http://www.ncbi.nlm.nih.gov/gene/7320> | UBE2B | 0.76 | 2 | 0.86 | 2 | 0.87 |
| <http://www.ncbi.nlm.nih.gov/gene/5889> | RAD51C | 0.67 | 2 | 0.77 | 2 | 0.88 |
| <http://www.ncbi.nlm.nih.gov/gene/1161> | ERCC8 | 0.69 | 2 | 0.78 | 2 | 0.88 |
| <http://www.ncbi.nlm.nih.gov/gene/1069> | CETN2 | 1.01 | 2 | 1.14 | 2 | 0.88 |
| <http://www.ncbi.nlm.nih.gov/gene/1111> | CHK1 | 0.59 | 2 | 0.66 | 2 | 0.88 |
| <http://www.ncbi.nlm.nih.gov/gene/3364> | HUS1 | 0.75 | 2 | 0.85 | 2 | 0.88 |
| <http://www.ncbi.nlm.nih.gov/gene/2140> | EYA3 | 0.54 | 10 | 0.61 | 6 | 0.89 |
| <http://www.ncbi.nlm.nih.gov/gene/8243> | SMC1A | 0.60 | 2 | 0.68 | 2 | 0.89 |
| <http://www.ncbi.nlm.nih.gov/gene/1643> | DDB2 | 0.88 | 2 | 0.99 | 2 | 0.89 |
| <http://www.ncbi.nlm.nih.gov/gene/64210> | MMS19 | 0.65 | 2 | 0.73 | 2 | 0.89 |
| <http://www.ncbi.nlm.nih.gov/gene/7161> | P73 | 0.70 | 2 | 0.78 | 2 | 0.90 |
| <http://www.ncbi.nlm.nih.gov/gene/64859> | SSB2 | 0.69 | 2 | 0.77 | 2 | 0.90 |
| <http://www.ncbi.nlm.nih.gov/gene/2073> | XPG | 0.66 | 2 | 0.74 | 2 | 0.90 |
| <http://www.ncbi.nlm.nih.gov/gene/675> | BRCA2 | 0.73 | 2 | 0.81 | 2 | 0.91 |
| <http://www.ncbi.nlm.nih.gov/gene/4595> | MUTYH | 0.85 | 6 | 0.93 | 6 | 0.91 |
| <http://www.ncbi.nlm.nih.gov/gene/79184> | BRCC3 | 0.75 | 2 | 0.82 | 2 | 0.91 |
| <http://www.ncbi.nlm.nih.gov/gene/641> | BLM | 0.78 | 2 | 0.85 | 2 | 0.91 |
| <http://www.ncbi.nlm.nih.gov/gene/1642> | DDB1 | 0.76 | 2 | 0.83 | 2 | 0.92 |
| <http://www.ncbi.nlm.nih.gov/gene/29935> | RPA4 | 0.82 | 2 | 0.89 | 2 | 0.92 |
| <http://www.ncbi.nlm.nih.gov/gene/4361> | MRE11 | 1.16 | 2 | 1.26 | 2 | 0.92 |
| <http://www.ncbi.nlm.nih.gov/gene/117283> | IP6K3 | 0.90 | 2 | 0.98 | 2 | 0.92 |
| <http://www.ncbi.nlm.nih.gov/gene/2138> | EYA1 | 0.62 | 2 | 0.67 | 2 | 0.92 |
| <http://www.ncbi.nlm.nih.gov/gene/3146> | HMGB1 | 0.65 | 2 | 0.70 | 2 | 0.92 |
| <http://www.ncbi.nlm.nih.gov/gene/1022> | CDK7 | 0.68 | 2 | 0.73 | 2 | 0.92 |
| <http://www.ncbi.nlm.nih.gov/gene/7515> | XRCC1 | 0.68 | 6 | 0.73 | 6 | 0.93 |
| <http://www.ncbi.nlm.nih.gov/gene/5111> | PCNA | 0.86 | 2 | 0.92 | 2 | 0.94 |
| <http://www.ncbi.nlm.nih.gov/gene/22909> | FAN1 | 0.83 | 2 | 0.89 | 2 | 0.94 |
| <http://www.ncbi.nlm.nih.gov/gene/27343> | POLL | 0.78 | 2 | 0.82 | 2 | 0.94 |
| <http://www.ncbi.nlm.nih.gov/gene/4683> | NBS1 | 1.01 | 2 | 1.06 | 2 | 0.96 |
| <http://www.ncbi.nlm.nih.gov/gene/11201> | POLI | 0.80 | 2 | 0.84 | 2 | 0.96 |
| <http://www.ncbi.nlm.nih.gov/gene/7157> | P53 | 0.90 | 2 | 0.93 | 2 | 0.96 |
| <http://www.ncbi.nlm.nih.gov/gene/546> | ATRX | 0.74 | 2 | 0.76 | 2 | 0.96 |
| <http://www.ncbi.nlm.nih.gov/gene/6996> | TDG | 0.71 | 2 | 0.73 | 2 | 0.97 |
| <http://www.ncbi.nlm.nih.gov/gene/201299> | RDM1 | 0.80 | 2 | 0.82 | 2 | 0.98 |
| <http://www.ncbi.nlm.nih.gov/gene/5884> | RAD17 | 0.69 | 6 | 0.70 | 6 | 0.98 |
| <http://www.ncbi.nlm.nih.gov/gene/27030> | MLH3 | 0.96 | 2 | 0.98 | 2 | 0.98 |
| <http://www.ncbi.nlm.nih.gov/gene/2068> | XPD | 0.81 | 2 | 0.82 | 2 | 0.98 |
| <http://www.ncbi.nlm.nih.gov/gene/3508> | IGHMBP2 | 0.91 | 2 | 0.92 | 2 | 0.98 |
| <http://www.ncbi.nlm.nih.gov/gene/23583> | UNG3 | 0.82 | 2 | 0.83 | 2 | 0.99 |
| <http://www.ncbi.nlm.nih.gov/gene/4255> | MGMT | 0.81 | 2 | 0.82 | 2 | 0.99 |
| <http://www.ncbi.nlm.nih.gov/gene/25> | ABL1 | 0.69 | 2 | 0.70 | 2 | 0.99 |
| <http://www.ncbi.nlm.nih.gov/gene/1854> | DUT | 0.99 | 2 | 0.99 | 2 | 0.99 |
| <http://www.ncbi.nlm.nih.gov/gene/10036> | CAF1 | 0.76 | 2 | 0.77 | 2 | 1.00 |
| <http://www.ncbi.nlm.nih.gov/gene/9156> | EXO1 | 0.81 | 2 | 0.81 | 2 | 1.00 |
| <http://www.ncbi.nlm.nih.gov/gene/7153> | TOP2 | 1.06 | 2 | 1.06 | 2 | 1.00 |
| <http://www.ncbi.nlm.nih.gov/gene/2237> | FEN1 | 0.77 | 2 | 0.76 | 2 | 1.00 |
| <http://www.ncbi.nlm.nih.gov/gene/8930> | MBD4 | 0.76 | 2 | 0.76 | 2 | 1.00 |
| <http://www.ncbi.nlm.nih.gov/gene/6117> | RPA1 | 0.66 | 2 | 0.65 | 2 | 1.01 |
| <http://www.ncbi.nlm.nih.gov/gene/2067> | ERCC1 | 0.82 | 2 | 0.81 | 2 | 1.01 |
| <http://www.ncbi.nlm.nih.gov/gene/10912> | GADD45G | 1.18 | 2 | 1.16 | 2 | 1.02 |
| <http://www.ncbi.nlm.nih.gov/gene/80198> | MUS81 | 0.66 | 2 | 0.65 | 2 | 1.03 |
| <http://www.ncbi.nlm.nih.gov/gene/7415> | P97 | 0.66 | 2 | 0.64 | 2 | 1.03 |
| <http://www.ncbi.nlm.nih.gov/gene/5965> | RECQL | 0.76 | 2 | 0.73 | 2 | 1.03 |
| <http://www.ncbi.nlm.nih.gov/gene/7141> | TNP1 | 0.62 | 2 | 0.60 | 2 | 1.04 |
| <http://www.ncbi.nlm.nih.gov/gene/5424> | POLD1 | 0.87 | 2 | 0.84 | 2 | 1.04 |
| <http://www.ncbi.nlm.nih.gov/gene/11144> | DMC1 | 0.73 | 2 | 0.70 | 2 | 1.04 |
| <http://www.ncbi.nlm.nih.gov/gene/165918> | RNF168 | 0.78 | 2 | 0.75 | 2 | 1.04 |
| <http://www.ncbi.nlm.nih.gov/gene/11232> | POLG2 | 0.89 | 2 | 0.84 | 2 | 1.05 |
| <http://www.ncbi.nlm.nih.gov/gene/7405> | UVRAG | 0.77 | 2 | 0.73 | 2 | 1.06 |
| <http://www.ncbi.nlm.nih.gov/gene/79661> | NEIL1 | 0.95 | 6 | 0.90 | 6 | 1.06 |
| <http://www.ncbi.nlm.nih.gov/gene/7158> | 53BP1 | 0.85 | 2 | 0.80 | 2 | 1.06 |
| <http://www.ncbi.nlm.nih.gov/gene/23411> | SIRT1 | 1.01 | 2 | 0.95 | 2 | 1.06 |
| <http://www.ncbi.nlm.nih.gov/gene/7507> | XPA | 1.05 | 2 | 0.99 | 2 | 1.06 |
| <http://www.ncbi.nlm.nih.gov/gene/5976> | UPF1 | 0.92 | 2 | 0.86 | 2 | 1.06 |
| <http://www.ncbi.nlm.nih.gov/gene/11219> | TREX2 | 0.78 | 2 | 0.73 | 2 | 1.07 |
| <http://www.ncbi.nlm.nih.gov/gene/4350> | MPG | 0.72 | 2 | 0.67 | 2 | 1.08 |
| <http://www.ncbi.nlm.nih.gov/gene/55215> | FANCI | 0.84 | 2 | 0.77 | 2 | 1.09 |
| <http://www.ncbi.nlm.nih.gov/gene/7520> | KU80 | 0.99 | 2 | 0.90 | 2 | 1.10 |
| <http://www.ncbi.nlm.nih.gov/gene/5582> | PRKCG | 0.99 | 2 | 0.90 | 2 | 1.10 |
| <http://www.ncbi.nlm.nih.gov/gene/142> | PARP1 | 0.92 | 2 | 0.83 | 2 | 1.11 |
| <http://www.ncbi.nlm.nih.gov/gene/51065> | RPS27L | 0.85 | 2 | 0.76 | 2 | 1.11 |
| <http://www.ncbi.nlm.nih.gov/gene/4869> | NPM1 | 0.96 | 2 | 0.86 | 2 | 1.11 |
| <http://www.ncbi.nlm.nih.gov/gene/8505> | PARG | 0.74 | 2 | 0.66 | 2 | 1.12 |
| <http://www.ncbi.nlm.nih.gov/gene/221120> | ALKBH3 | 0.90 | 2 | 0.79 | 2 | 1.13 |
| <http://www.ncbi.nlm.nih.gov/gene/11200> | CHK2 | 0.91 | 2 | 0.79 | 2 | 1.15 |
| <http://www.ncbi.nlm.nih.gov/gene/548593> | SLX1 | 0.96 | 10 | 0.83 | 6 | 1.16 |
| <http://www.ncbi.nlm.nih.gov/gene/25988> | HINFP | 0.94 | 2 | 0.81 | 2 | 1.17 |
| <http://www.ncbi.nlm.nih.gov/gene/84268> | RPAIN | 0.98 | 2 | 0.83 | 2 | 1.17 |
| <http://www.ncbi.nlm.nih.gov/gene/545> | ATR | 0.83 | 2 | 0.70 | 2 | 1.18 |
| <http://www.ncbi.nlm.nih.gov/gene/113510> | HELQ | 1.17 | 2 | 0.99 | 2 | 1.18 |
| <http://www.ncbi.nlm.nih.gov/gene/5378> | PMS1 | 1.09 | 2 | 0.92 | 2 | 1.18 |
| <http://www.ncbi.nlm.nih.gov/gene/7832> | BTG2 | 1.04 | 2 | 0.88 | 2 | 1.19 |
| <http://www.ncbi.nlm.nih.gov/gene/7508> | XPC | 1.04 | 6 | 0.88 | 6 | 1.19 |
| <http://www.ncbi.nlm.nih.gov/gene/84126> | ATRIP | 0.90 | 2 | 0.75 | 2 | 1.19 |
| <http://www.ncbi.nlm.nih.gov/gene/79677> | SMC6 | 1.26 | 2 | 1.05 | 2 | 1.20 |
| <http://www.ncbi.nlm.nih.gov/gene/25788> | RAD54B | 0.97 | 2 | 0.80 | 2 | 1.21 |
| <http://www.ncbi.nlm.nih.gov/gene/9031> | BAZ1B | 0.90 | 10 | 0.74 | 6 | 1.21 |
| <http://www.ncbi.nlm.nih.gov/gene/3978> | LIG1 | 0.86 | 2 | 0.71 | 2 | 1.21 |
| <http://www.ncbi.nlm.nih.gov/gene/1386> | ATF2 | 0.78 | 2 | 0.64 | 2 | 1.22 |
| <http://www.ncbi.nlm.nih.gov/gene/83990> | BRIP1 | 0.90 | 2 | 0.73 | 2 | 1.22 |
| <http://www.ncbi.nlm.nih.gov/gene/51720> | RAP80 | 1.02 | 2 | 0.83 | 2 | 1.23 |
| <http://www.ncbi.nlm.nih.gov/gene/9319> | TRIP13 | 1.03 | 2 | 0.83 | 2 | 1.23 |
| <http://www.ncbi.nlm.nih.gov/gene/5893> | RAD52 | 0.78 | 2 | 0.64 | 2 | 1.23 |
| <http://www.ncbi.nlm.nih.gov/gene/11284> | PNKP | 0.82 | 2 | 0.66 | 2 | 1.24 |
| <http://www.ncbi.nlm.nih.gov/gene/5426> | POLE | 0.83 | 2 | 0.66 | 2 | 1.25 |
| <http://www.ncbi.nlm.nih.gov/gene/672> | BRCA1 | 1.03 | 2 | 0.82 | 2 | 1.26 |
| <http://www.ncbi.nlm.nih.gov/gene/10721> | POLQ | 1.04 | 2 | 0.82 | 2 | 1.26 |
| <http://www.ncbi.nlm.nih.gov/gene/7298> | TYMS | 1.12 | 2 | 0.89 | 2 | 1.26 |
| <http://www.ncbi.nlm.nih.gov/gene/146956> | EME1 | 0.92 | 2 | 0.73 | 2 | 1.26 |
| <http://www.ncbi.nlm.nih.gov/gene/1196> | CLK2 | 0.88 | 2 | 0.70 | 2 | 1.27 |
| <http://www.ncbi.nlm.nih.gov/gene/5810> | RAD1 | 0.97 | 2 | 0.76 | 2 | 1.28 |
| <http://www.ncbi.nlm.nih.gov/gene/472> | ATM | 1.13 | 2 | 0.88 | 2 | 1.29 |
| <http://www.ncbi.nlm.nih.gov/gene/79840> | XLF | 1.05 | 2 | 0.82 | 2 | 1.29 |
| <http://www.ncbi.nlm.nih.gov/gene/1647> | GADD45A | 1.19 | 2 | 0.92 | 2 | 1.29 |
| <http://www.ncbi.nlm.nih.gov/gene/4331> | MNAT1 | 1.21 | 2 | 0.92 | 2 | 1.31 |
| <http://www.ncbi.nlm.nih.gov/gene/7335> | UBE2V1 | 1.17 | 2 | 0.89 | 2 | 1.31 |
| <http://www.ncbi.nlm.nih.gov/gene/2074> | CSB | 0.79 | 2 | 0.60 | 2 | 1.32 |
| <http://www.ncbi.nlm.nih.gov/gene/7518> | XRCC4 | 0.87 | 2 | 0.65 | 2 | 1.33 |
| <http://www.ncbi.nlm.nih.gov/gene/55120> | FANCL | 1.04 | 2 | 0.78 | 2 | 1.34 |
| <http://www.ncbi.nlm.nih.gov/gene/8846> | ALKBH1 | 1.10 | 2 | 0.82 | 2 | 1.34 |
| <http://www.ncbi.nlm.nih.gov/gene/23626> | SPO11 | 0.83 | 2 | 0.62 | 2 | 1.34 |
| <http://www.ncbi.nlm.nih.gov/gene/1786> | DNMT1 | 0.85 | 2 | 0.63 | 2 | 1.35 |
| <http://www.ncbi.nlm.nih.gov/gene/5883> | RAD9 | 1.10 | 2 | 0.81 | 2 | 1.35 |
| <http://www.ncbi.nlm.nih.gov/gene/84142> | ABRA1 | 1.11 | 2 | 0.81 | 2 | 1.36 |
| <http://www.ncbi.nlm.nih.gov/gene/5888> | RAD51 | 1.02 | 2 | 0.74 | 2 | 1.37 |
| <http://www.ncbi.nlm.nih.gov/gene/11277> | TREX1 | 1.06 | 2 | 0.76 | 2 | 1.39 |
| <http://www.ncbi.nlm.nih.gov/gene/5187> | PER1 | 1.23 | 2 | 0.88 | 2 | 1.40 |
| <http://www.ncbi.nlm.nih.gov/gene/29883> | CAF1 | 1.03 | 2 | 0.73 | 2 | 1.41 |
| <http://www.ncbi.nlm.nih.gov/gene/6917> | TF2S | 1.42 | 2 | 1.00 | 2 | 1.42 |
| <http://www.ncbi.nlm.nih.gov/gene/3981> | LIG4 | 0.98 | 2 | 0.68 | 2 | 1.44 |
| <http://www.ncbi.nlm.nih.gov/gene/9656> | MDC1 | 1.14 | 2 | 0.77 | 2 | 1.47 |
| <http://www.ncbi.nlm.nih.gov/gene/121642> | ALKBH2 | 1.20 | 2 | 0.79 | 2 | 1.52 |
| <http://www.ncbi.nlm.nih.gov/gene/6241> | RRM2 | 0.34 | 2 | 0.21 | 2 | 1.61 |
| <http://www.ncbi.nlm.nih.gov/gene/1663> | DDX11 | 1.16 | 2 | 0.70 | 2 | 1.66 |
