# Supplementary figures and images for: DNA Damage Response Factors from Diverse Pathways, Including DNA Crosslink Repair, Mediate Alternative End Joining
Source: PLoS Genet. 2015 Jan 28;11(1):e1004943. doi: 10.1371/journal.pgen.1004943 (PMC4309583; doi:10.1371/journal.pgen.1004943)

**A**

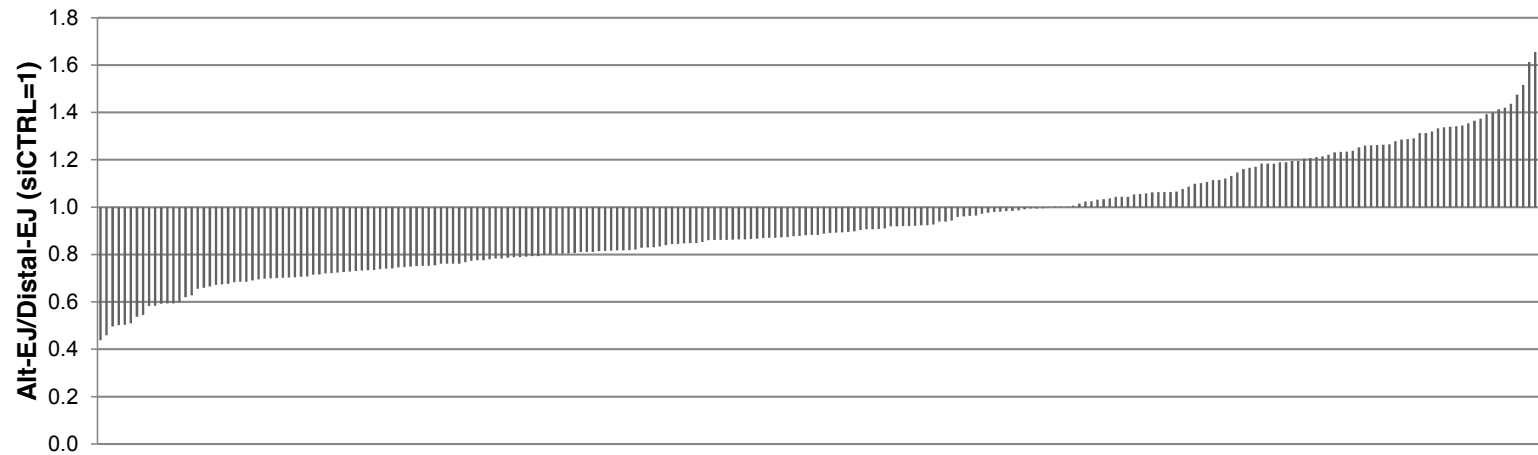

**B**

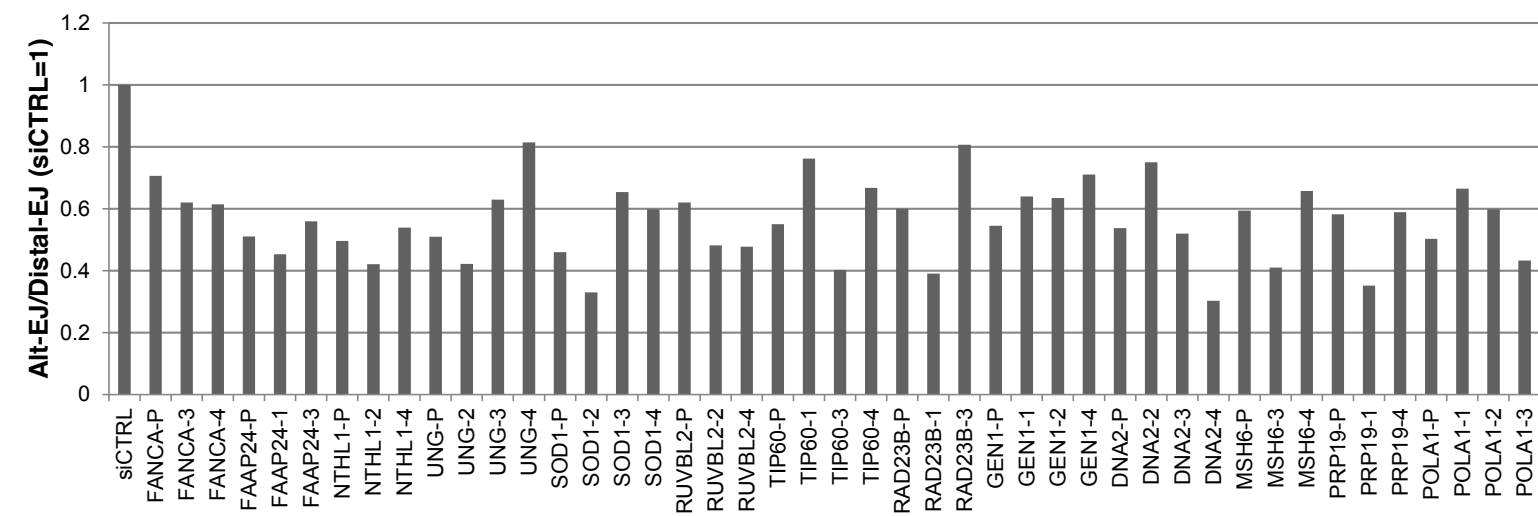

Supplement: S1 Fig — (A) for each siRNA in the screen (S2 Table), and (B) for each siRNA shown in Fig. 1C.Since each fold change is calculated relative to siCTRL, the ratio for siCTRL = 1. (PDF) [file pgen.1004943.s003.pdf]

Supplemental Figure S2

**A**

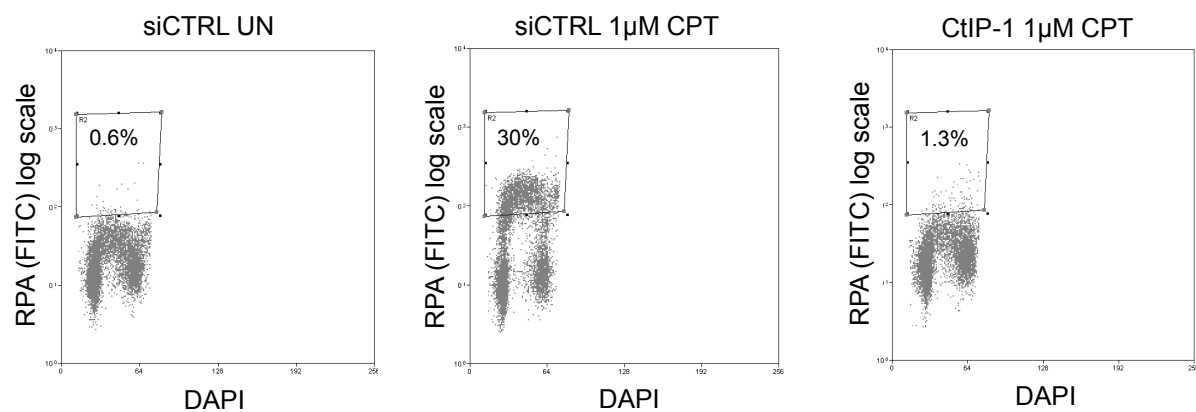

**B**

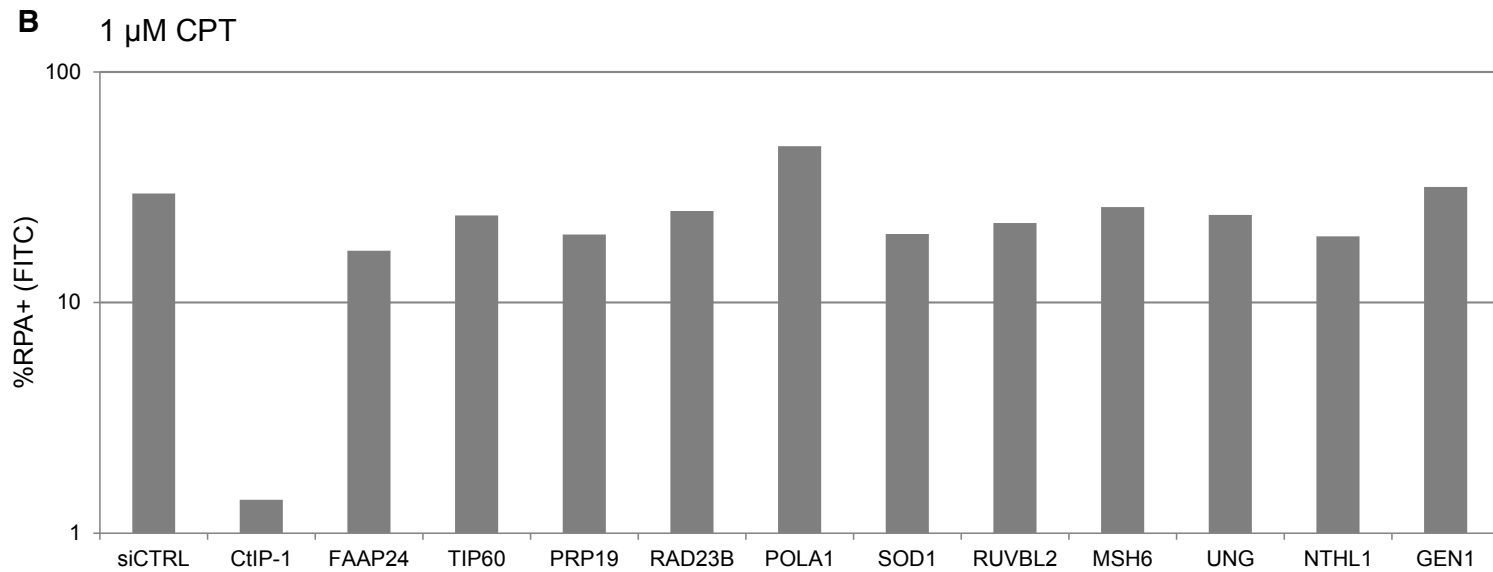

Supplement: S2 Fig — (A) Shown are representative flow cytometry plots from cells treated with siCTRL without camptothecin (siCTRL UN), siCTRL with camptothecin (siCTRL 1μM CPT) and siCtIP with camptothecin (CtIP-1 1μM CPT). U2OS cells were treated and analyzed for RPA staining as described in Fig. 6E. (B) Analysis of the effects of siRNA treatment on end resection. Shown is the percentage of cells with RPA staining after siRNA treatment using siRNA pools targeting the genes shown (i.e. the genes from Fig. 2C, except DNA2 and FANCA, which are shown in Fig. 6F). (PDF) [file pgen.1004943.s004.pdf]

Supplementary Figure S3

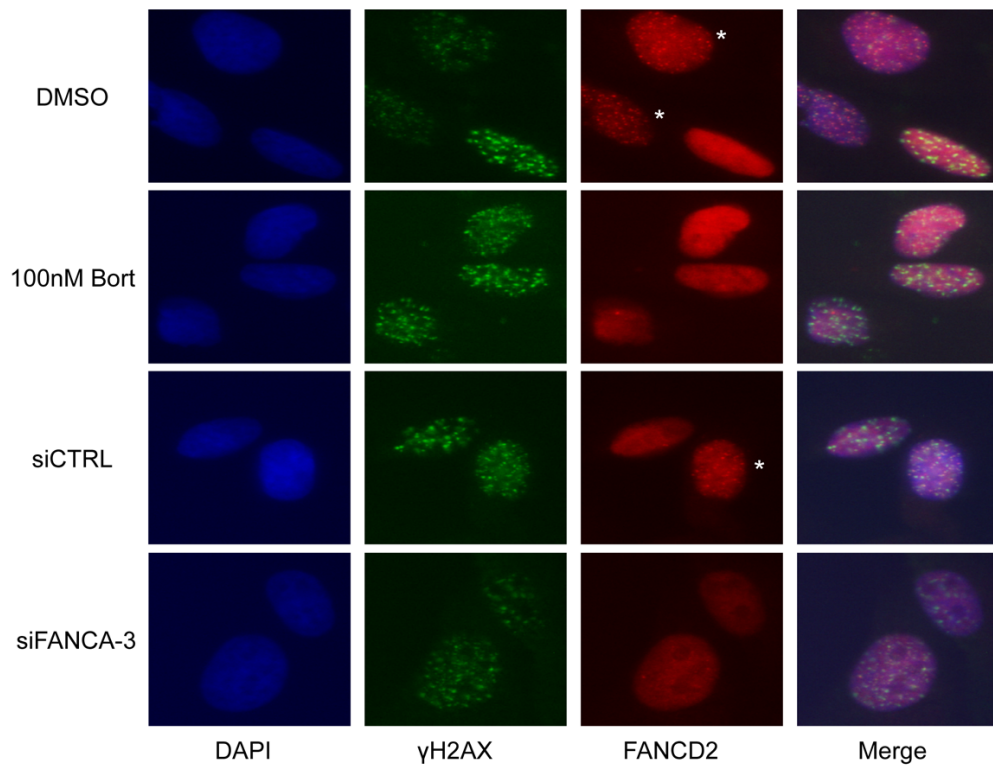

Supplement: S3 Fig — Cells were treated with 10 Gy IR and allowed to recover (4 hr) prior to fixation and immunostaining for FANCD2 and the DSB marker γH2AX. *indicates representative cells with >20 FANCD2 foci. (PDF) [file pgen.1004943.s005.pdf]
